# Heavy metals in lake surface sediments in protected areas in Poland: concentration, pollution, ecological risk, sources and spatial distribution

**DOI:** 10.1038/s41598-022-19298-y

**Published:** 2022-09-02

**Authors:** Mariusz Sojka, Joanna Jaskuła, Jan Barabach, Mariusz Ptak, Senlin Zhu

**Affiliations:** 1 Department of Land Improvement, Environmental Development and Spatial Management, Poznań University of Life Sciences, Piątkowska 94E, 60‐649 Poznan, Poland; 2 Department of Hydrology and Water Management, Adam Mickiewicz University, Krygowskiego 10, 61-680 Poznan, Poland; 3 College of Hydraulic Science and Engineering, Yangzhou University, Yangzhou, 225009 China

**Keywords:** Environmental sciences, Hydrology, Limnology

## Abstract

This paper presents the state and spatial distribution of surface sediment contamination of 77 lakes in Poland by Cr, Ni, Cd, Pb, Zn, and Cu. The analyzed lakes were located within a network of nature protection areas in the territory of the European Union (EU). Spatial distribution of the heavy metals (HMs), factors favoring the delivery/accumulation of HMs in surface sediments, and pollution sources were analyzed. The results indicate the contamination of lake sediments by HMs, but the potentially toxic effects of HMs are only found in single lakes. The spatial distribution of Cr indicates predominant impacts of point sources, while for Pb, Ni, and Zn, the impact of non-point sources. The analysis showed the presence of areas with very high values of particular HMs (hot spots) in the western part of Poland, while a group of 5 lakes with very low values of Ni, Pb, and Zn (cold spots) was identified in the central part of Poland. Principal component analysis showed that presence of wetlands is a factor limiting HMs inflow to lakes. Also, lower HMs concentrations were found in lake surface sediments located in catchments with a higher proportion of national parks and nature reserves. Higher HMs concentrations were found in lakes with a high proportion of Special Protection Areas designated under the EU Birds Directive. The positive matrix factorization analysis identified four sources of HMs. High values of HMs concentrations indicate their delivery from industrial, urbanized, and agricultural areas. However, these impacts overlap, which disturbs the characteristic quantitative profiles assigned to these pollution sources.

## Introduction

Heavy metals (HMs) are considered as one of the most problematic environmental pollutants^1,2^. It is linked to their persistence, non-biodegradability, toxicity, and bioaccumulation. Their origin in water bodies may be either natural (e.g. weathering of bedrock, volcanic eruptions) or anthropogenic (e.g. metal ores and coal mining, coal burning, industry, urbanization, sewage treatment, fertilization, atmospheric deposition from fossil fuel combustion, deforestation, tourism, aquaculture, fishery)^3–10^. Dominant sources of HMs differ at the continental scale. In Africa, the dominant source of HMs is bedrock weathering, in North America mining and industry, and in Asia and Europe domestic wastewaters^11^.

According to the latest study results, sediments are dominated by elements primarily originating from anthropogenic sources^12–14^. Many studies show that industrial sources generally have the greatest impact on HMs pollution^15^. In urban lakes, the dominant sources are urban and industrial effluents; in rural areas, lakes are mainly supplied with agricultural runoff and domestic wastes^13^. Lakes in protected areas are also exposed to the supply of HMs from anthropogenic sources^16–18^. It results from the fact that these lakes’ whose catchments are located beyond the protected areas. According to Cuculić et al. (2009), HMs in lake sediments in national parks are both of natural and anthropogenic origin. In lakes isolated from pollution sources, the pattern of HMs concentration is different than that in lakes subject to human pressure^19^. Research conducted by Vukosav et al.^20^ showed that lake sediments in the Plitvice Lakes National Park (Croatia) are generally not polluted with HMs. Also, sediment contamination has not been reported in Smolensk Lake National Park^21^. According to the research by Sojka et al.^22^, even in lakes with catchments located entirely within the Bory Tucholskie National Park (Poland), higher concentrations of HMs in sediments were observed as a result of dry and wet deposition from the atmosphere.

There are abundant study results presenting the state of HMs pollution of sediments around the globe^23–26^. Researches are usually conducted for the surface layer of sediments^27–29^. Their primary purpose is to capture the effects of point and non-point pollution sources, but also the assessment of the effect of hydraulic processes at the river–lake interface and morphometric conditions on the distribution of HMs in the water body^30,31^. Temporal and spatial variability of HMs in lakes sediments is usually determined by many overlapping chemical, physical, and biological processes. The pollution is largely variable between lakes as well as within lakes^31,32^. The spatial distribution of pollutants is modified by hydrological and hydrodynamic conditions that affect water and sediment transport, as well as the exchange of HMs between water and sediments^33,34^. Sediments deposited in lakes provide a unique record of the processes occurring in particular lakes and catchment areas^35,36^.

Natura 2000 (N2) is the newest form of nature conservation established in Poland in 2004. N2 areas were formed in all Member States of the European Union establishing the European Ecological Network Natura 2000. At present, the N2 network covers almost 20% of the country's area in Poland. The lakes included in the N2 area are those that have been subjected to anthropogenic pressure over the years, but also currently receive pollution delivered from the catchment area. This raises the question of what their pollution is and what factors determine this pollution. To answer the above questions, in this study, we (1) analyzed the concentration of HMs in lake surface sediments, (2) assessed the contamination of lake surface sediments by HMs and their toxic effects on aquatic biota, (3) determined the spatial pattern of HMs concentrations in lake surface sediments, (4) identified the sources of HMs delivery to lakes, and (5) exposed the factors promoting and or limiting HMs delivery and accumulation in lake surface sediments.

## Study site description

**Figure 1:**
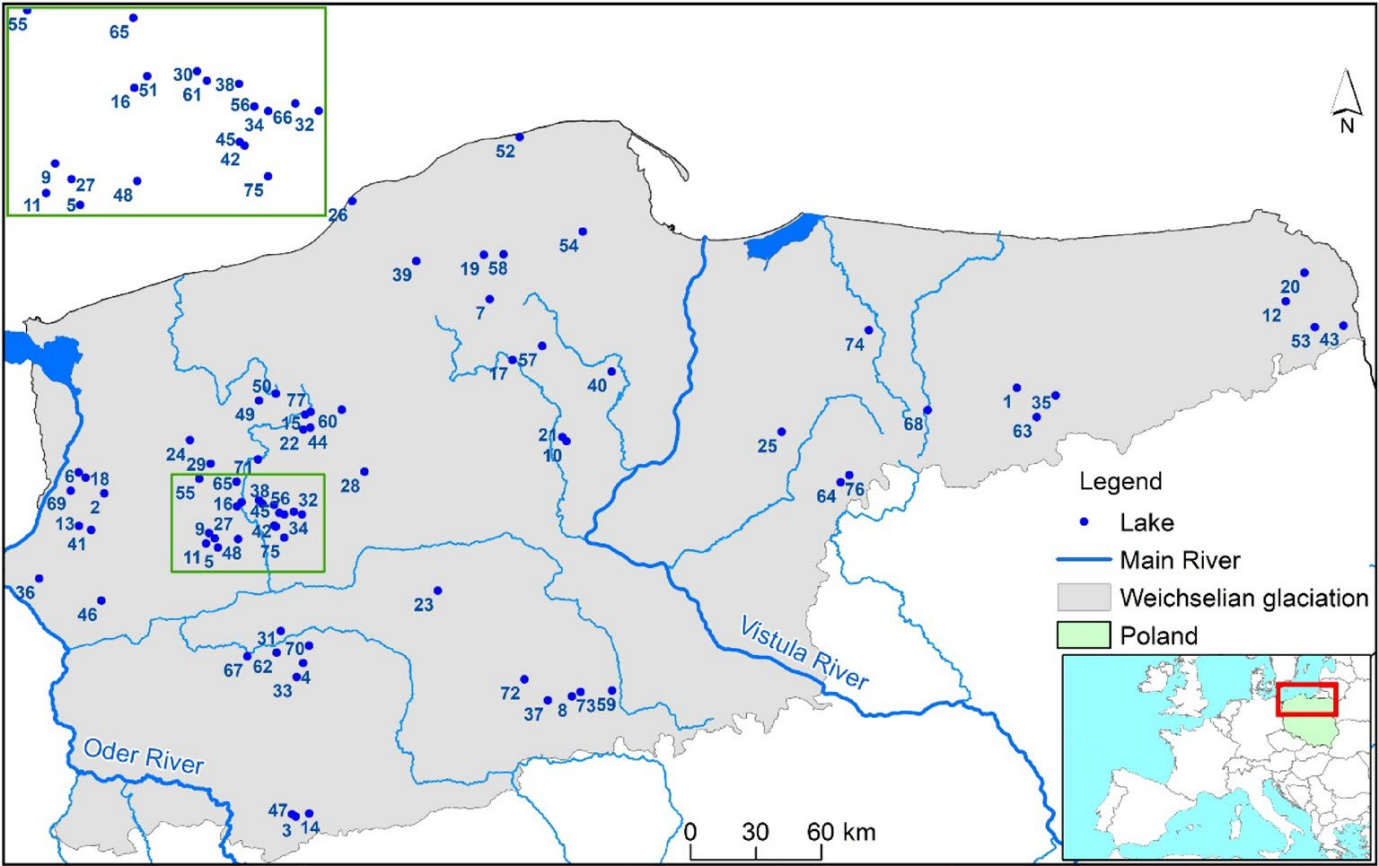
Study site location (the figure was generated in ArcMap 10.7, https://desktop.arcgis.com/en/arcmap/).

There are more than 7000 lakes in Poland. The vast majority of them are located in the northern part of the country. They include exceptionally environmentally valuable objects requiring detailed monitoring and protection^37^. There are 146 and 791 lakes located in nature reserve areas and landscape park areas respectively^38^. In this paper, the analysis covers 77 lakes located in the N2 areas (Fig. 1) (Suppl. Table S1). The genesis of the lakes analyzed in the paper is primarily postglacial. Their morphometric properties are therefore highly variable. This fact considerably affects the course of processes and phenomena that occur in them, shaping their abiotic as well as biotic properties. This statement can be referred to conditions concerning water exchange, groundwater supply, etc. For example, the deepest of the analyzed lakes (Drawsko (15), Wukśniki (74), Babięty Wielkie (1), etc.) are in contact with geological formations with different physical and chemical properties (among others, sands and gravels, loams, peats). The complexity of the functioning of these ecosystems is magnified by the relations between the lake and its catchment, including those approximate to quasi-natural (dominance of forest areas) and those strongly affected by human pressure.

## Materials and methods

### Chemical data

The results of the chemical analysis of HMs provided by the Chief Inspectorate of Environmental Protection (CIEP) in Poland were used in this study. The study material is the result of HMs determinations from a 5 cm surface layer of sediments sampled from the deepest point in the lake. Sediments for analyses were collected using a Van Veen sampler. The elements selected for the analysis included Cd, Cr, Cu, Ni, Pb, and Zn. The HMs concentration in sediment samples was determined using inductively coupled plasma–optical emission spectrophotometry (ICP-OES). The limits of detection for Cd, Cr, Cu, Ni, Pb, and Zn were 0.05, 0.30, 0.40, 0.40, 1.0 and 0.5 mg^.^kg^−1^, respectively.

CIEP conducts cyclical research on surface sediments of lakes in the scope of the national environmental monitoring aimed at the determination of the content of HMs. In 2020, the research covered 160 lakes. The basic criterion of lakes’ selection for the analysis was their location within the N2 protected areas and within the boundaries of the Weichselian glaciation. Based on the aforementioned criteria, a total of 77 lakes were selected for analysis. Another criterion of lake selection was the availability of morphometric data. The analysis of archival materials provided by the Inland Fisheries Institute in Olsztyn showed that the data were not available only for Kosino (27), Piaseczno (41), Rudno (51), and Sianowskie (54) lakes. Therefore, these lakes were partly excluded from the principal component analysis.

### Data and spatial analysis

The identification of lakes within the range of the N2 protected areas employed the geodatabase of the General Directorate for Environmental Protection (GDEP) in Poland. The GDEP geodatabase includes information on the spatial extent of protected areas across Poland, i.e. N2 areas (including special areas of conservation—“habitats” N2h and special protection areas—“Bird” N2b), as well as national parks (NP), landscape parks (LP), and nature reserves (NR). The total share of protected areas was determined for the individual lake catchments (PrA). During the analysis, data presenting the land use pattern in the catchments were used, i.e. the proportion of artificial areas (ArA), agricultural areas (AgA), forest and semi-natural areas (FoA), wetland areas (WeA), and water bodies (WaA). Land use structure was determined based on the Corine Land Cover geodatabase provided by the European Protection Agency. Moreover, for a description of the water and matter dynamics in the catchment, the river network density was determined. Moreover, several characteristics were developed for the lakes concerning hydrological type (flow-through (FT), exorheic (Ex) and endorheic lakes (En)), surface area (LA), volume (LV), average (AvD), maximum depth (MaD), and shoreline development index (SDI). To assess the impact of the catchment on the lake, the lake drainage ratio (LDR) was calculated. The ratio of catchment area to the lake area is a commonly used indicator of the effect of catchment processes on lake dynamics^39^. For each of the lakes, average water exchange time (WET) was determined as the ratio of the lake volume and water outflow from the catchment. The methods of calculation of particular characteristics are presented in Suppl. Table S2.

### Analysis of pollution of surface sediments and ecotoxicity

The assessment of the lakes’ sediment pollution involved the calculation of the geoaccumulation index (*I*_*geo*_)^40^, contamination factor (CF)^41^, enrichment factor (EF)^42^, pollution load index (PLI)^43^, and metal pollution index (MPI)^44^. The indices *I*_*geo*_, CF, and EF permit the assessment of sediment pollution for a single element. PLI and MPI allowed for integrated (complex) assessment of sediment pollution for all HMs. The calculation of the values of *I*_*geo*_, CF, EF, and PLI employed values of the geochemical background proposed by Tylmann et al.^45^. The values of the geochemical background are as follows: Cd 0.5 mg kg^−1^; Cr 5 mg kg^−1^; Ni 5 mg kg^−1^; Cu 6 mg kg^−1^; Pb 10 mg kg^−1^; and Zn 48 mg kg^−1^. Moreover, the calculation of EF employed the value of the geochemical background for Fe (10,000 mg kg^−1^ in this study). Values of the geochemical background and Fe concentrations in a sample of lake sediments permitted standardization of the results^46^. The methods used to calculate geochemical indices and the related classes of sediment pollution with HMs are presented in Suppl. Table S3.

The assessment of the potential toxic effect of HMs was conducted using the toxic risk index (TRI)^47^. The TRI values were calculated based on the threshold (TEC) and probable (PEC) effect concentrations of HMs. Values of PEC and TEC were proposed by MacDonald et al.^48^. The TEC values for Cd, Cr, Cu, Ni, Pb, and Zn are 0.99, 43, 32, 23, 36, and 120 mg·kg^−1^, respectively. PEC values are as follows for Cd, Cr, Cu, Ni, Pb, and Zn: 5.0, 110, 150, 49, 130, and 460 mg kg^−1^, respectively. The method of calculation of the TRI and toxicity class is presented in Suppl. Table S3.

### Statistical analysis

The statistical analysis involved the determination of descriptive statistics for the analyzed HMs. The minimum, mean, median, and maximum values were determined. Moreover, for each of the elements, values of Q1 and Q3 quartiles, as well as the interquartile range (IQR), were determined. For the presentation of the variability of concentrations of particular HMs in surface sediments, combined violin and boxplot diagrams were developed. In addition, a Spearman's rank correlation analysis was conducted to assess the HMs pattern in lake sediments. The verification of whether HMs concentrations have normal distribution employed the Shapiro–Wilk test. To determine whether differences between concentrations of particular HMs in sediments are significant, the non-parametric Wilcoxon rank test for pairs was applied. Before commencing multidimensional analyses, i.e. cluster analysis (CA) and principal component analysis (PCA), at the first stage, the dataset was analyzed in terms of occurrence of outliers using the Grubbs test. Due to the right-skewed distribution of the analyzed HMs, a one-tailed Grubbs test was applied. The identified outliers were replaced with values 1% higher than the maximum value that was not recognized as an outlier. The essence of this operation was on the one hand limiting the effect of outliers on the analysis result, and on the other hand, ensuring reduction of loss of data during the CA and PCA.

For identifying similar groups of lakes by HMs concentrations in surface sediments, CA was applied. CA was performed using the Ward method, with Euclidean distance squared as the measurement of similarity. The delimitation of groups and sub-groups was performed by adopting the following cut-off thresholds: 66% and 33%, respectively for groups and sub-groups^49^. For comparison of HMs concentration values in the designated groups and sub-groups, the Kruskal–Wallis test and Mann–Whitney U test were applied. Moreover, for the designated CA groups and sub-groups of lakes, indices characterizing their catchments were compared in terms of land use, development of the hydrographic network, dynamics of water exchange, and lake morphometric parameters. The detailed methods of calculation of particular indices and scope of source materials are provided in Suppl. Table S1.

For analysis of autocorrelations between HMs concentrations in the analyzed lakes, the Global Moran's I and Getis-Ord Gi* statistics were applied. The analysis of spatial autocorrelation primarily aimed at determining whether the analyzed HMs concentrations have random distribution (null hypothesis), or either clusters or dispersion occur. In the case of occurrence of a cluster structure, proof exists of the occurrence of natural or anthropogenic spatial processes determining HMs concentrations in surface sediments of lakes. For the identification of clusters of lakes with high (hot-spot) and low (cold-spot) HMs concentrations, the Getis-Ord GI* statistic was applied.

In the final stage, a detailed analysis of HMs sources was performed using principal component analysis (PCA). The PCA was performed for all HMs against the background of environmental variables describing lake catchment and lake morphometry. The following group of environmental parameters was distinguished for lake catchment: area (CA), the share of artificial areas (ArA), the share of agricultural areas (AgA), the share of forest and seminatural areas (FoA), the share of wetland (WeA) and water bodies (WaA), drainage density (DrD), and the share of protected areas in the catchment (PrA). The following parameters were distinguished for lakes: longitude (Lon), latitude (Lat), lake area (LA), volume (LV), average depth (AvD), maximum depth (MaD), hydrological type (flow-through (FT), exorheic (Ex) and endorheic lakes (En), shoreline development index (SDI) and lake drainage ratio (LDR). The lake hydrologic types during the PCA were assigned the following values of FT—3, En—2, and Ex—1. PCA was performed in three different variants. Due to the right-skewed distribution of HMs, they were transformed using the Box-Cox method before PCA. The Box-Cox (1964)^50^ transformation is used to transform variables so that their distribution after transformation is as approximate to normal as possible. The formula of the Box-Cox transformation is as follows:

$$\left\{ {\begin{array}{*{20}l} {\frac{{\left( {c_{i} + \alpha } \right)^{\lambda } - 1}}{\lambda },} \hfill & {\quad {\text{if}}\quad \lambda \ne 0} \hfill \\ {{\text{log}}\left( {c_{i} + \alpha } \right),} \hfill & {\quad {\text{if}}\quad \lambda = 0} \hfill \\ \end{array} } \right.$$

where *c*_*i*_ means the original variable, *c*_*i*_*’* is a transformed variable, *λ* means the primary transformation parameter and adopts values in a range from − 5 to 5, and *α* is a parameter of moving variable. Finally, the transformed concentration values obtained as a result of the Box-Cox transformation were subject to scaling in a range from 0 to 1. The procedure aimed at avoiding the situation where elements with the highest values have the greatest impact on the analysis result.

By the Kaiser criterion, the principal components were significant when eigenvalues were higher than 1. Moreover, it was assumed that when factor loadings between the HMs or environmental parameters and selected principal components were in a range of 0.75–1.00, 0.50–0.75, and 0.30–0.50, they were strongly, moderately, and weakly correlated, respectively^51^.

The positive matrix factorization (PMF) was used for HMs source apportionment in lake sediments^52^. The PMF model can be expressed as^53^:

$${x}_{i,j}= \sum\limits_{p=1}^{n}{g}_{i,p}{f}_{p,j}+{e}_{i,j}$$

where *p* is the number of pollution sources (factors), *g* is the mass of HMs contributed by each factor to each sample, *f* is the HMs profile of each pollution source, and *e* is the residual. Contributions of pollution sources and HMs profiles are derived by minimizing the objective function *Q*:

$$Q= \sum_{i=1}^{n}\sum_{j=1}^{m}{\left(\frac{{x}_{i,j}-{\sum }_{p=1}^{n}{g}_{i,p}{f}_{p,j}}{{u}_{i,j}}\right)}^{2}$$

where *u*_*i,j*_ is the uncertainty. The number of significant factors was determined according to the approach presented by Comero et al.^54^. Optimum numbers of pollution sources (factors) were found by the analyzing *Q* value^55^.

To identify the uncertainty *u*_*i,j*_ for individual HMs, the methods described by Polissar et al.^56^, Pandolfi et al.^57^ and Luo et al.^58^ were used:

For the HMs concentrations below the detection limit:

$${x}_{i,j}=\frac{{d}_{i,j}}{2}, {u}_{i,j}=\frac{{5d}_{i,j}}{6}$$

For the HMs concentrations above the detection limit:

$$x_{i,j} = c_{i,j} ,\quad {\text{if}}\quad x_{i,j} \le 3d_{i,j} ,\,\,u_{i,j} = \frac{1}{3}d_{i,j} + 0.2 \cdot c_{i,j}$$

$$x_{i,j} = c_{i,j} \quad {\text{if}}\quad x_{i,j} > 3d_{i,j} ,\,\,\,u_{i,j} = \frac{1}{3}d_{i,j} + 0.1 \cdot c_{i,j}$$

where *x*_*i,j*_ is the concentration used in the PMF model, *d*_*i,j*_ is the detection limit of each component, *u*_*i,j*_ is the uncertainty of *x*_*i,j*_, *c*_*i,j*_ is the measured HM concentration.

The statistical analyses were performed using programs Statistica 13.1, Canoco 5.0, and R. All statistical analyses were performed at a significance level of 0.05. Moreover, the PMF model version 5.0 developed by the Environmental Protection Agency (EPA), US was used^53^.

## Results

### Variability of HM concentrations in surface sediments of lakes

Values of the descriptive statistics for HMs concentrations in surface sediments of 77 lakes located in the protected N2 areas are presented in Table 1. The analysis of median and mean concentration values of HMs showed that they can be arranged in the ascending order Cd < Ni < Cr < Cu < Pb < Zn. Concentrations of particular HMs differ significantly at a level of 0.05, considering the results of the Wilcoxon rank-test for pairs.

**Table 1:** Values of the descriptive statistics of HMs concentrations (mg^.^kg^−1^) in surface sediments of lakes in the N2 areas.

Statistics	Cr	Ni	Cu	Zn	Cd	Pb
Minimum	2.51	1.33	0.20	13.0	0.025	2.0
1st Quartile	6.50	4.83	7.15	44.0	0.117	22.0
Mean	13.06	9.58	21.66	87.5	0.961	45.7
Median	9.44	7.24	13.30	72.0	0.486	37.0
3rd Quartile	17.50	12.80	24.00	126.0	1.590	70.0
Maximum	106.00	29.00	164.00	240.0	7.630	150.0
Standard deviation	13.15	6.25	25.71	53.9	1.164	33.8
Interquartile range	11.0	7.97	16.85	82.0	1.473	48.0
Coefficient of variation	100.67	65.26	118.69	61.53	121.19	74.08
Skewness (-)	4.90	1.13	3.24	0.98	2.76	0.96
Kurtosis (-)	32.80	0.73	13.59	0.19	12.99	0.50
Distribution type (-)	Lognormal	Lognormal	Lognormal	Lognormal	Lognormal	Lognormal
Number of outliers (-)	2	0	4	0	1	0

**Figure 2:**
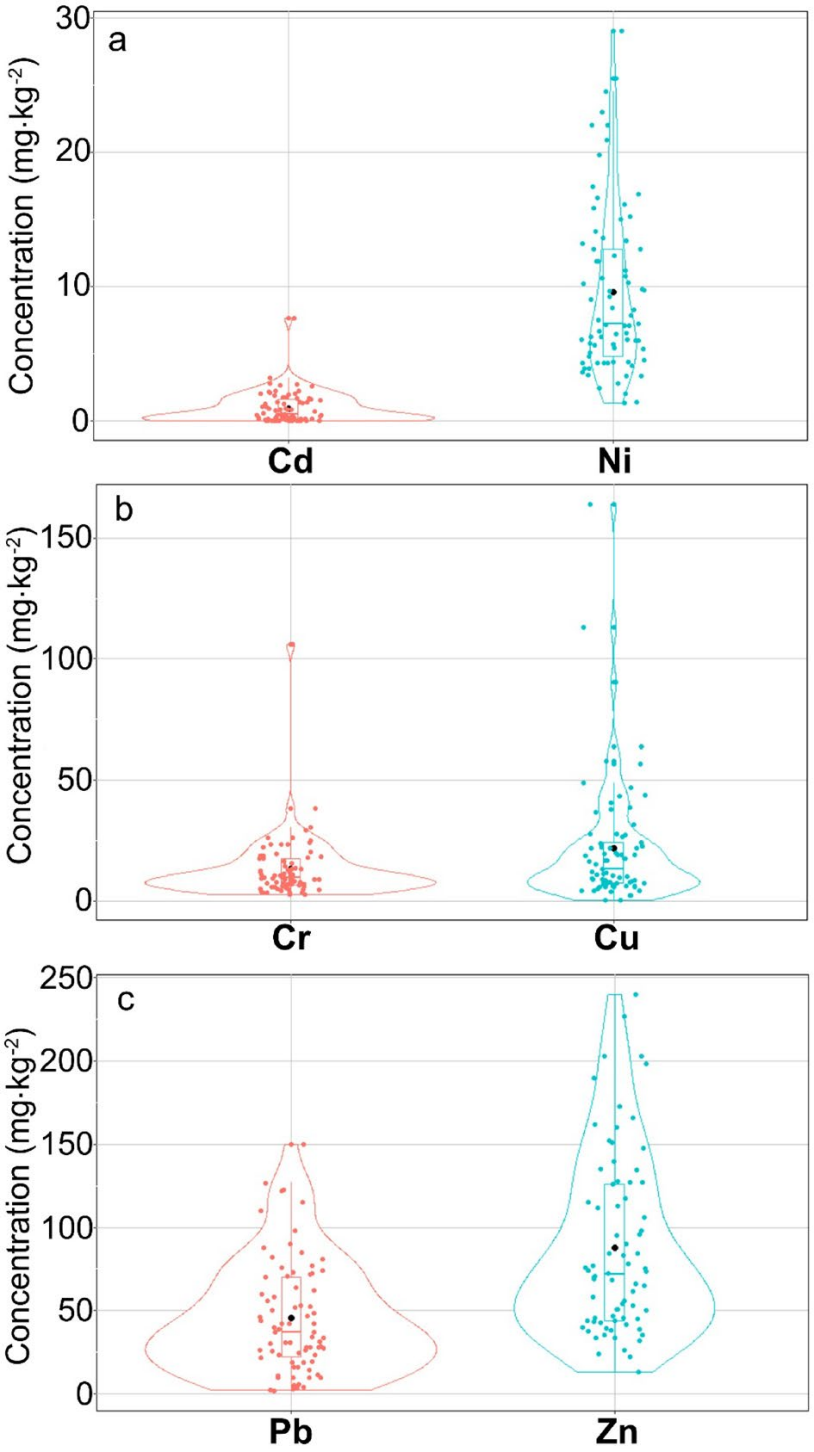
Variability of heavy metal concentrations in surface sediments of lakes in N2 areas: Cd and Ni (**a**), Cr and Cu (**b**), as well as Pb and Zn (**c**).

It was also observed that for all HMs, mean values are higher than the median values, suggesting a right-skewed distribution. This is also confirmed by the skewness statistic, which showed a value higher than 0 for all HMs (from 0.96 to 4.9). Values of the kurtosis statistic were at a level from 0.19 to 32.80. This suggests that the distributions of HMs are leptokurtic – slim/pointed. Finally, the results of the Shapiro–Wilk test showed that in all HMs, the distributions differ from normal distribution at a significance level of 0.05. For the presentation of the variability of particular HMs in lake surface sediments, combined violin and boxplot diagrams were developed (Fig. 2). Graphical presentation of the results confirms the large variation of HMs in lake sediments.

Data analysis using the Grubbs-Beck test showed that Cr, Cu, and Cd concentrations include outliers. Particularly, Cr, Cu, and Cd datasets include 2, 4, and 1 outlier, respectively. The analysis showed that Cr outliers occurred in Lakes Bierzwnik (5) and Piaseczno Duze (42), Cu in Lakes Dlugie Wigierskie (12), Kamienny Most (24), Lubosz Wielki (33), and Wielkie (70), and Cd in Lake Trzebun (65).

### Pollution of surface sediments

**Figure 3:**
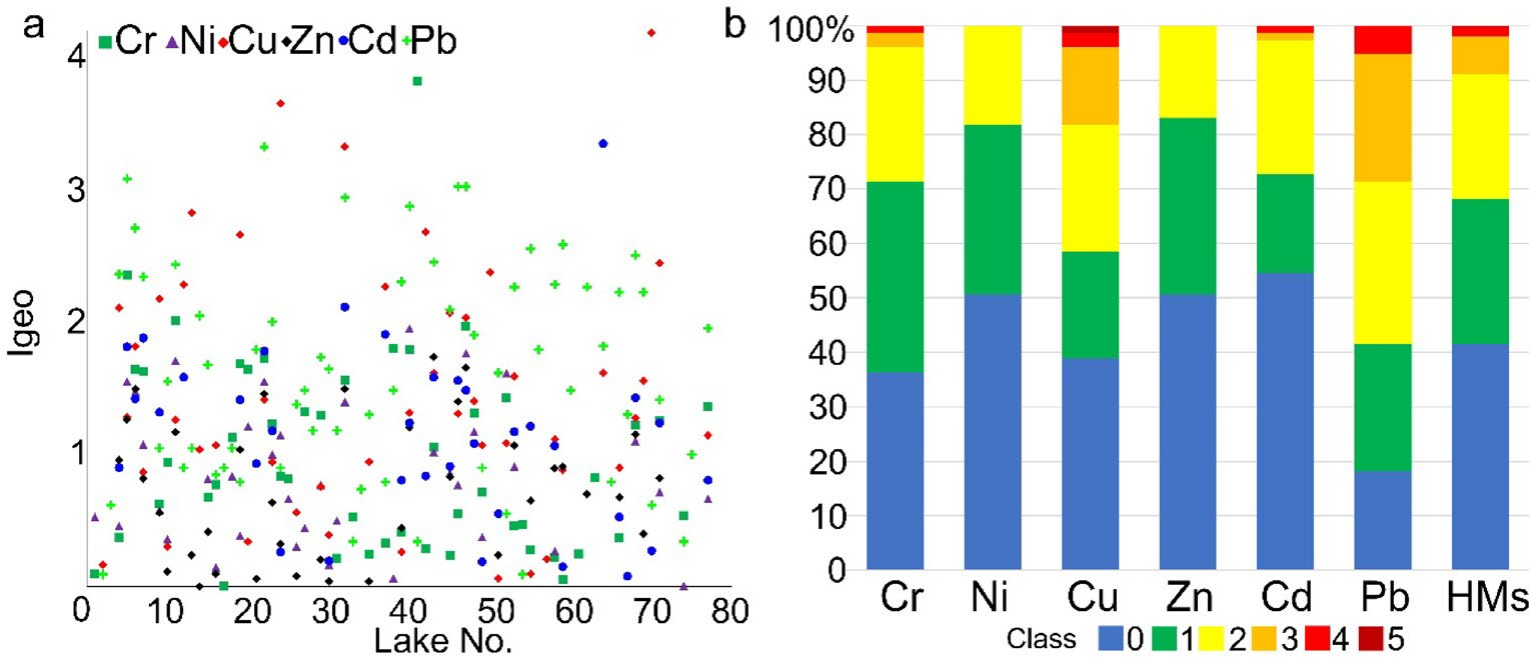
*I*_*geo*_ index values higher than 0 (**a**) with the classification of the state of lake sediment pollution (**b**).

The analysis of HMs pollution of lake surface sediments based on the *I*_*geo*_ showed that the greatest pollution concerns Cu and Pb. In the case of Cu and Pb, *I*_*geo*_ values higher than 2 occurred in 14 and 22 lakes, respectively (Fig. 3a), which suggests that the sediments are contaminated above moderate levels. Moreover, for Cu, the *I*_*geo*_ value was higher than 4, which indicates that the surface sediment is heavily contaminated. The sediments were least polluted with Ni and Zn. In more than 50% of samples, *I*_*geo*_ values did not exceed 0 (class 0 and 1). The percentage share of lakes in particular pollution classes for the *I*_*geo*_ index is presented in Fig. 3b. In the case of 5 lakes—Białkowskie (4), Bierzwnik (5), Chłopowo (11), Postne (46), and Przytoń (49)—*I*_*geo*_ index values were higher than 2 for two elements, and for Lake Lubosz Wielki (32) for as many as three elements. In each of the discussed lakes, the observed *I*_*geo*_ index values were higher than 2 for Pb, and additionally in four cases for Cu, in two cases for Cr, and one case for Cd.

**Figure 4:**
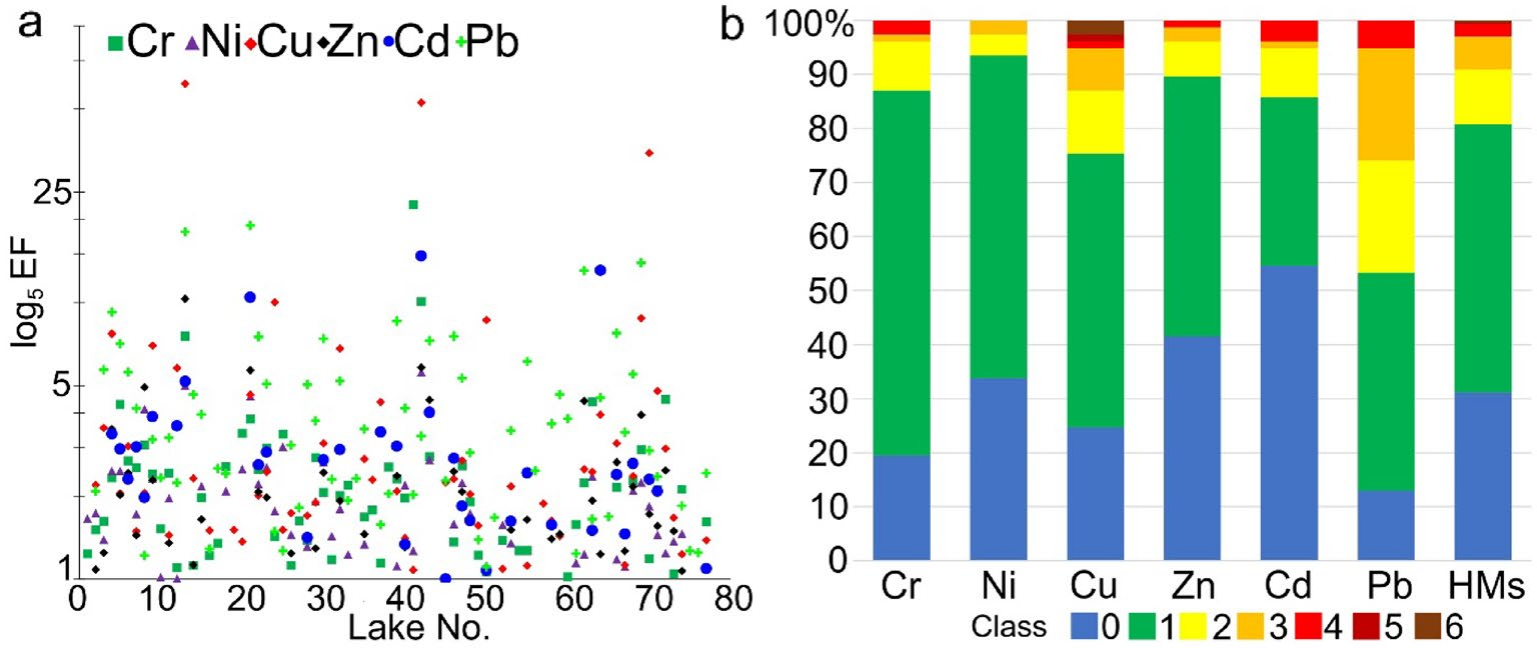
EF index values higher than 1 (**a**) with the classification of the state of pollution of surface sediments (**b**).

Somewhat different results were obtained by analyzing EF index values and referring them to the threshold values adopted for pollution classes of sediments. In the case of 29 lakes, pollution of surface sediments was at a very low level. For all elements, EF values were below 3. In a further 20 lakes, EF values for all elements were below 5, suggesting slight enrichment. A further 19 lakes showed EF values for single elements in a range from 5 to 10, pointing to moderately intensive sediment pollution. In reference to 6 lakes, EF values for single elements varied from 10 to 25 (Fig. 4a). For 3 lakes, EF values for single elements were higher than 25. Considering single elements, EF values in the majority of cases were lower than 3 (Fig. 4b). EF values higher than 10 occurred in sporadic cases for Cr (2), Cu (4), Zn (1), Cd (3), and Pb (4). The surface sediments were generally more polluted with Pb and Cu, and less with Ni and Zn.

**Figure 5:**
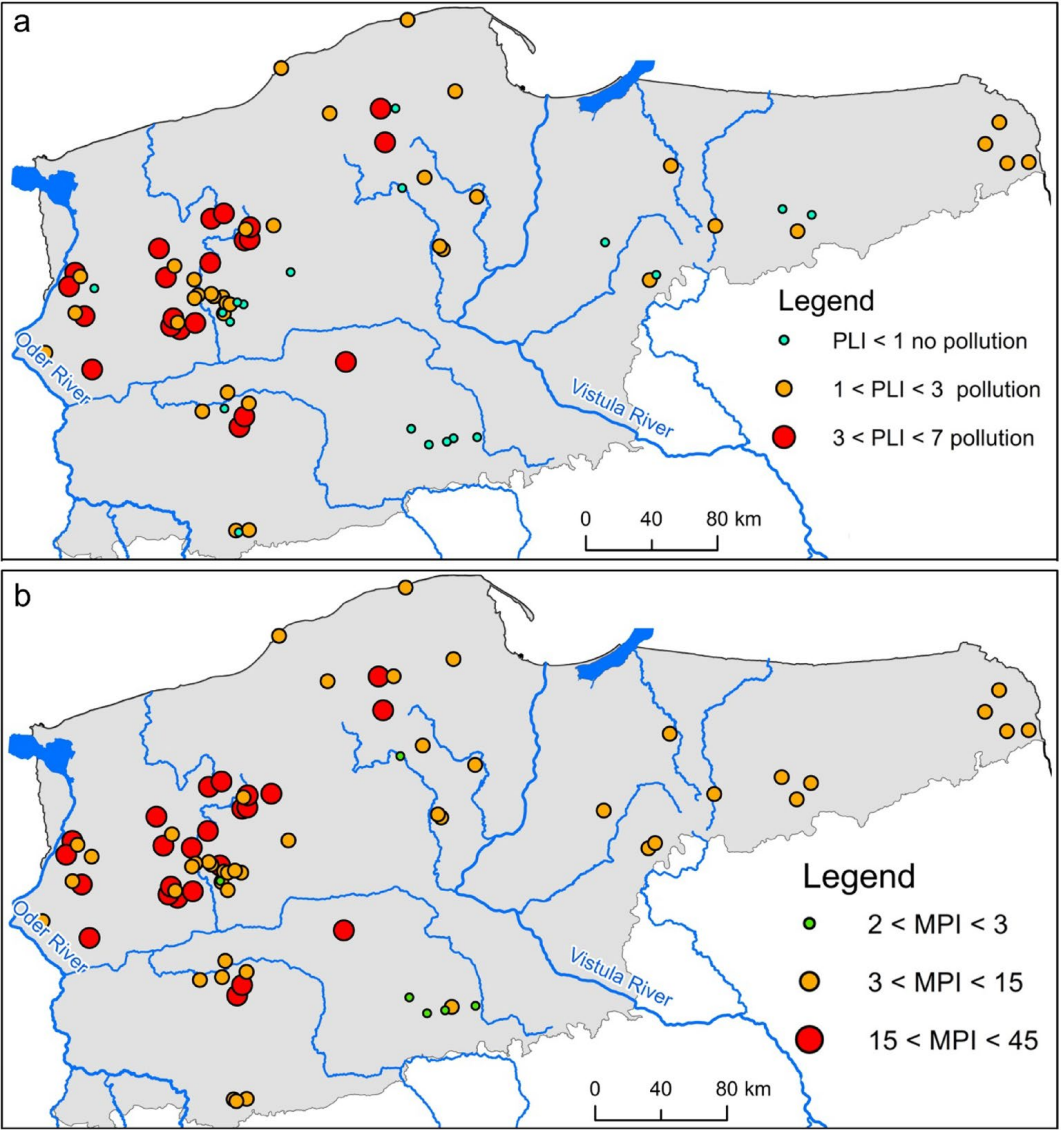
State of HM pollution of lake surface sediments based on PLI (**a**) and MPI (**b**) (the figure was generated in ArcMap 10.7, https://desktop.arcgis.com/en/arcmap/).

Considering PLI values permitted complex assessment of pollution of sediments. PLI values were generally within a range from 0.40 to 6.60, averaging 2.17. It was observed that for 19 lakes, the PLI values are below 1, indicating a lack of pollution of sediments with HMs. The location of these lakes is presented by means of green circles (Fig. 5a).

The highest PLI values, exceeding 4, occurred in Lakes Bierzwnik (5), Binowskie (6), Boruja Duża (7), Kaleńskie (22), Lubosz Wielki (33), Piaseczno (41), Pławno (44), Przytoczno (48), Przytoń (49), and Wełtyńskie (69). A similar pattern of pollution of lakes was obtained using the MPI values (Fig. 5b). MPI values for the analyzed lakes were in a range from 2.28 to 37.95, averaging 12.48. The highest MPI values occurred in Lakes Bierzwnik (5), Kaleńskie (22), Lubosz Wielki (33), and Przytoń (49), and the lowest MPI values, below 3, for Lakes Budzisławskie (8), Dobrzyk (17), Niedzięgiel (37), Płociczno (45), Skulska Wieś (59), and Wierzbiczańskie (72).

### Ecotoxicity of HMs

**Figure 6:**
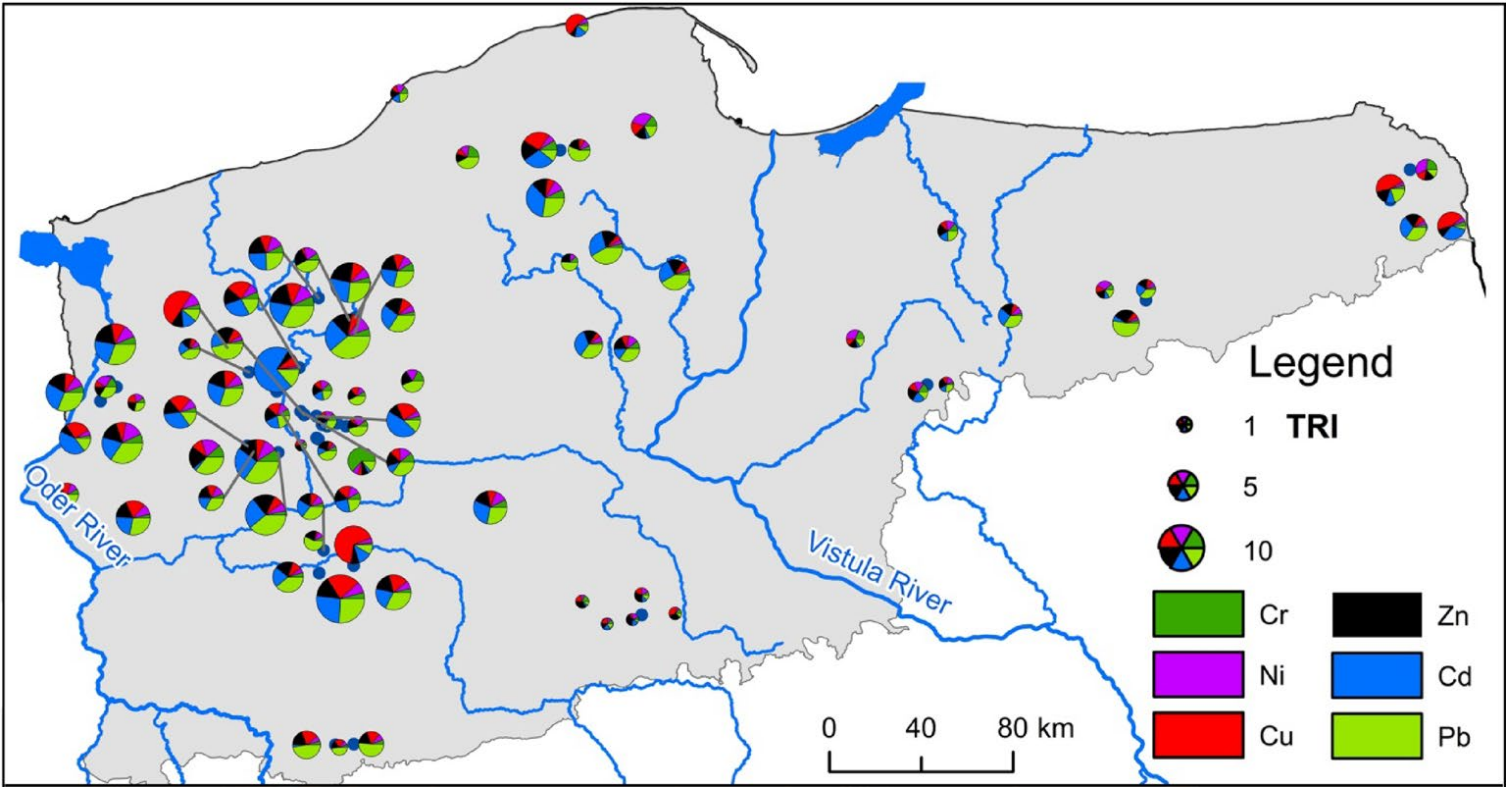
Potential ecotoxic effect of HMs accumulated in surface sediments of lakes (the figure was generated in ArcMap 10.7, https://desktop.arcgis.com/en/arcmap/).

The analysis of the potential toxic effect of particular HMs based on the Sediment Quality Guidelines showed concentrations higher than PEC values in only three cases. In single samples, concentrations of Cu, Cd, and Pb higher than PEC values were recorded in Lakes Wielkie (70), Trzebuń (65), and Kaleńskie (22), respectively. Concentration values in a range from midpoint effect concentration (MEC) to probable effect concentration (PEC) occurred once for Cr, Cu, and Cd. In the case of Pb, values in a range from 83 to 130 (Level 3) occurred 9 times. The obtained results suggest that the potential ecotoxic effect usually occurs for single elements, and concerns a small number of lakes. Considering the total ecotoxic effect of all HMs accumulated in surface sediments based on the TRI, no threat is recorded in 64 cases. In 13 cases, TRI values vary from 5 to 10, indicating minor toxic effects (Fig. 6).

### Spatial variability of HMs concentrations

**Figure 7:**
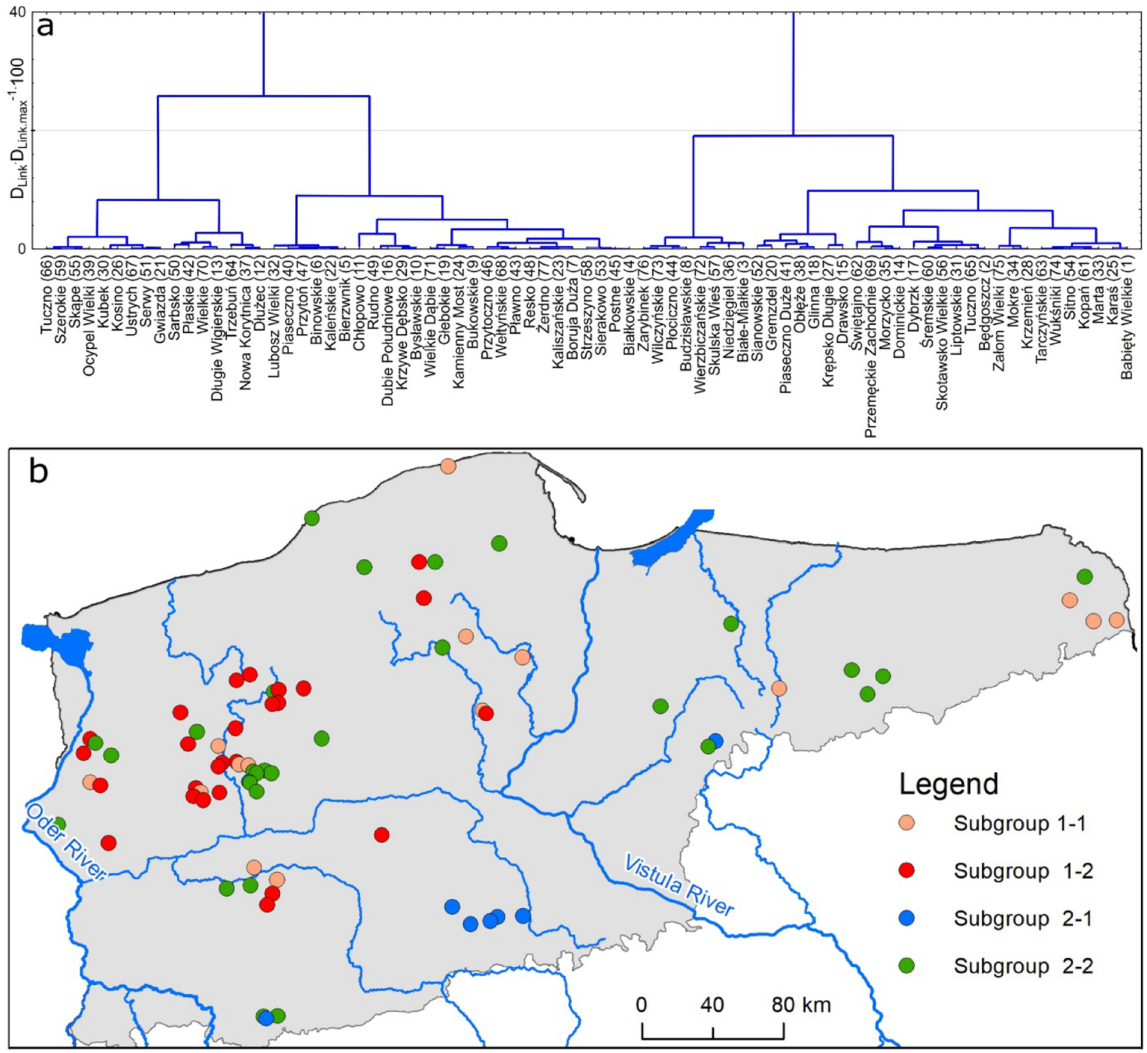
Results of the CA: division into groups of lakes by similarity of HM concentrations (**a**) and graphical presentation (**b**) [the (**b**) was generated in ArcMap 10.7, https://desktop.arcgis.com/en/arcmap/].

The CA permitted a division of the lakes into two groups. In each of the groups, two sub-groups were designated, in terms of occurrence of HMs in their sediments (Fig. 7a). Group I included a total of 42 lakes, whereas sub-groups 1–1 and 1–2 included 16 and 26 lakes, respectively. Group 2 is somewhat smaller, including 35 lakes: 8 lakes in sub-group 2–1 and 27 lakes in sub-group 2–2.

The resulting groups are characterized by variability of the concentration of particular HMs, as confirmed by the results of the Kruskal–Wallis test. For all HMs, significant differences occurred in reference to the median values. Pair comparisons by means of the Mann–Whitney U test for all the analyzed HMs allow their concentrations in particular sub-groups to be ordered as follows: Cr (2–1 < 1–1 < 2–2 < 1–2), Ni (2–1 < 2–2 = 1–1 < 1–2), Cu (2–1 = 2–2 < 1–1 = 1–2), Zn (2–1 < 2–2 < 1–1 < 1–2), Cd (2–2 = 2–1 < 1–1 = 1–2), and Pb (2–1 < 2–2 = 1–1 < 1–2). The obtained results show that in terms of all analyzed HMs, sediments of lakes classified in sub-group 2–1 were the least polluted. The highest level of pollution was recorded for sediments of lakes included in sub-group 1–2. Results of the CA, although helpful in a broader analysis of the state of pollution of lakes, provide no information on the spatial relations between the analyzed lakes, for the identification of local factors affecting HMs concentrations in sediments. Due to this, the obtained division into groups and sub-groups is presented graphically (Fig. 7b). The map reveals the random character of pollution of lakes with HMs. To confirm the results, analysis was performed using the Global Moran's I statistic and Getis-Ord Gi* statistic in the environment of the ArcMap software, separately for each element. For the Global Moran's I statistic, the null hypothesis states that the elements are randomly distributed. When the p-value is less than 0.05, the null hypothesis can be rejected. Results of analyses using the Moran's I statistic are presented in Table 2.

**Table 2:** Results of autocorrelation analysis using Global Moran's I statistic.

Parameters	Moran I		
	*P* value	z score	Pattern
Cd	0.204	1.271	Random
Cr	0.000	− 3.845	Dispersed
Cu	0.140	1.475	Random
Pb	0.007	2.701	Clustered
Ni	0.001	3.207	Clustered
Zn	0.000	3.746	Clustered

The p values were lower than 0.05 and positive z-score values were obtained for Pb, Ni, and Zn. This means the spatial distribution of high and/or low concentrations in the dataset is more spatially clustered than would be expected if the underlying spatial processes were random. In the case of Cr, p values lower than 0.05 and negative z-score values were also obtained, suggesting that the spatial distribution of high and low concentrations is more spatially dispersed than would be expected if the underlying spatial processes were random. For Cd and Cu, p values were higher than 0.05. This shows that concentrations of these elements in sediments are of random distribution.

**Figure 8:**
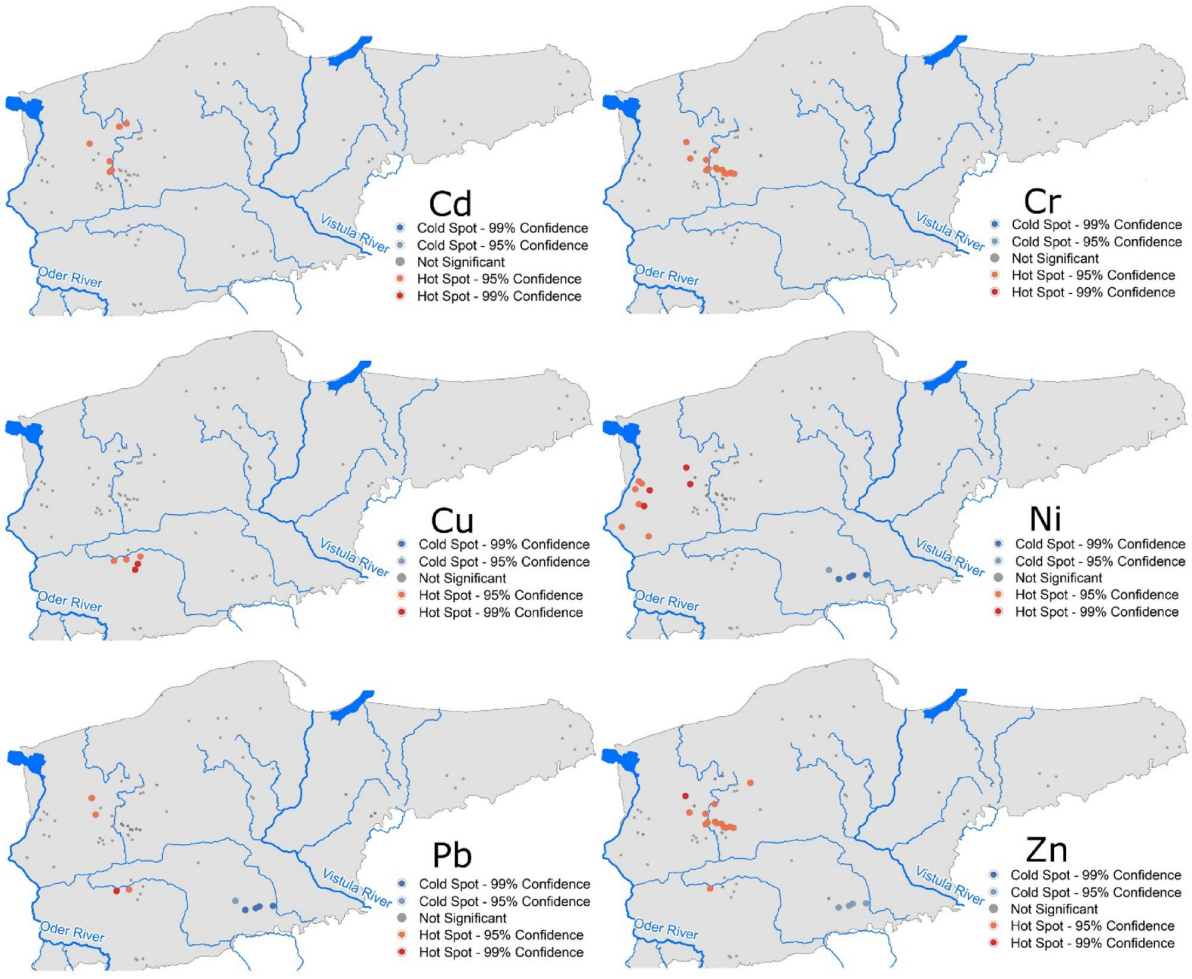
Occurrence of hot and cold spots in lakes in terms of the content of particular HMs in surface sediments (the figure was generated in ArcMap 10.7, https://desktop.arcgis.com/en/arcmap/).

Analysis using the Getis-Ord Gi* statistic identified both cold and hot spots (Fig. 8). For Cr, the occurrence of a cluster of lakes was recorded with high concentrations of the element in sediments. This concerned Lakes Resko (50), Przytoń (49), Kamienny Most (24), Trzebuń (65), Rudno (51), and Dubie Południowe (16). The spatial analysis using the Getis-Ord Gi* statistic allowed identification of a group of 13 lakes with higher than average Cr content. This group is located in the north-western part of Poland, and includes the following lakes: Kamienny Most (24), Wielkie Dąbie (71), Sierakowo (55), Trzebuń (65), Krzywe Dębsko (30), Rudno (51), Szerokie (61), Nowa Korytnica (38), Dubie Południowe (16), Tuczno (66), Sitno (56), Liptowskie (32), and Marta (34). The group of lakes with increased Cu concentrations in sediments is located in the central-western part of Poland and includes 5 lakes: Wielkie (70), Śremskie (62), Tuczno (67), Białkowskie (4), and Lubosz Wielki (33). For Ni, two groups of lakes were designated, those with low and high concentrations. The former group includes Lakes Wierzbiczańskie, Skulska Wieś, Wilczyńskie, Budzisławskie, and Niedzięgiel. The group of lakes with higher than average Ni values includes 10 lakes in the western and north-western Poland (Kamienny Most (24), Binowskie (6), Glinna (18), Sierakowo (55), Wełtyńskie (69), Będgoszcz (2), Dłużec (13), Piaseczno (41), Morzycko (36), and Postne (46)). In reference to lakes with low Pb concentration values, in their sediments, their location overlaps with that designated for Ni (5 lakes: Wierzbiczańskie (72), Skulska Wieś (59), Wilczyńskie (73), Budzisławskie (8), and Niedzięgiel (37)). Moreover, two small groups of lakes were designated with high Pb values in sediments, namely Lakes Kamienny Most (24) and Sierakowo (55), as well as Śremskie (62) and Tuczno (67). In the case of Zn, like for Ni and Pb, the occurrence of groups with both high and low values was observed. Higher than average values of Zn and Cr, and partially Cd, usually occurred in the overlapping groups of lakes. Low Ni, Pb, and Zn concentrations also occurred in the same group of lakes in the central part of Poland.

**Figure 9:**
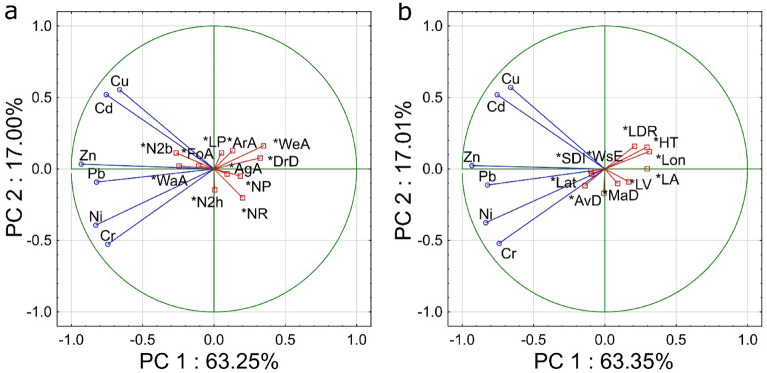
Results of PCA against parameters describing the lake catchment (**a**) and parameters describing the lake (**b**).

Identification of pollution sources and factors contributing to HMs accumulation in lake surface sediments was performed by PCA. The analysis was performed considering additional variables characterizing the lake catchment and variables describing the lake in terms of its location, morphometric parameters, and hydrological types. The results allowed us to distinguish two factors in both cases. In the first case, the analysis was performed against variables describing catchment areas, where all lakes were considered. The results indicate that factors PC1 and PC2 explain 63.25% and 17.00% of the internal structure of the data, respectively. All HMs were strongly negatively correlated with PC 1, while Cu and Cd were positively correlated with PC 2, and Cr was negatively correlated with PC 2 at a moderate level. Among the variables describing the lake catchment, drainage density (DrD) and wetland area (WeA) were weakly positively correlated with PC 1. The results indicate that Cr, Ni, Pb, and Cd concentrations are lower in the sediments of lake catchments with higher drainage density (DrD). On the other hand, lower concentrations of Cr, Ni, Zn, and Pb are observed in the surface sediments of lake catchments with higher wetland areas (WeA), while higher concentrations of Cd and Zn are found in lake sediments with increasing surface water area in the catchment (WaA). Moreover, it was found that a higher proportion of national parks (NP) and nature reserves (NR) in the catchment corresponds to lower concentrations of Zn and Pb, and Cu, Zn, and Cd in lakes, respectively (Fig. 9a). It was observed that higher concentrations of Zn, Cd, and Pb are observed in lake surface sediments with an increase in the catchment areas within the range of N2b. Moreover, the proportion of natural parks (LP) and N2h areas is not related to the HM concentrations in lake sediments. In LP and N2h areas, surface waters are not the object of conservation. Similar ordinations of HMs were obtained by performing PCA against the variables describing the lake properties (Fig. 9b). The differences are due to the fact that 4 lakes for which there was a lack of morphometric data were omitted during this analysis. The results indicate that in general, lakes in western Poland had higher concentrations of HMs in surface sediments. This is confirmed by the negative correlation of HMs concentrations with longitude (Lon). In addition, the surface sediments of larger lakes are generally less contaminated with HMs. This is due to limited sediment transport within the lake. In addition, in flow-through (FT) lakes and lakes with larger drainage ratios (LDR), lower Cr and Ni concentrations were observed. This indicates the delivery of these elements directly from the catchment.

**Figure 10:**
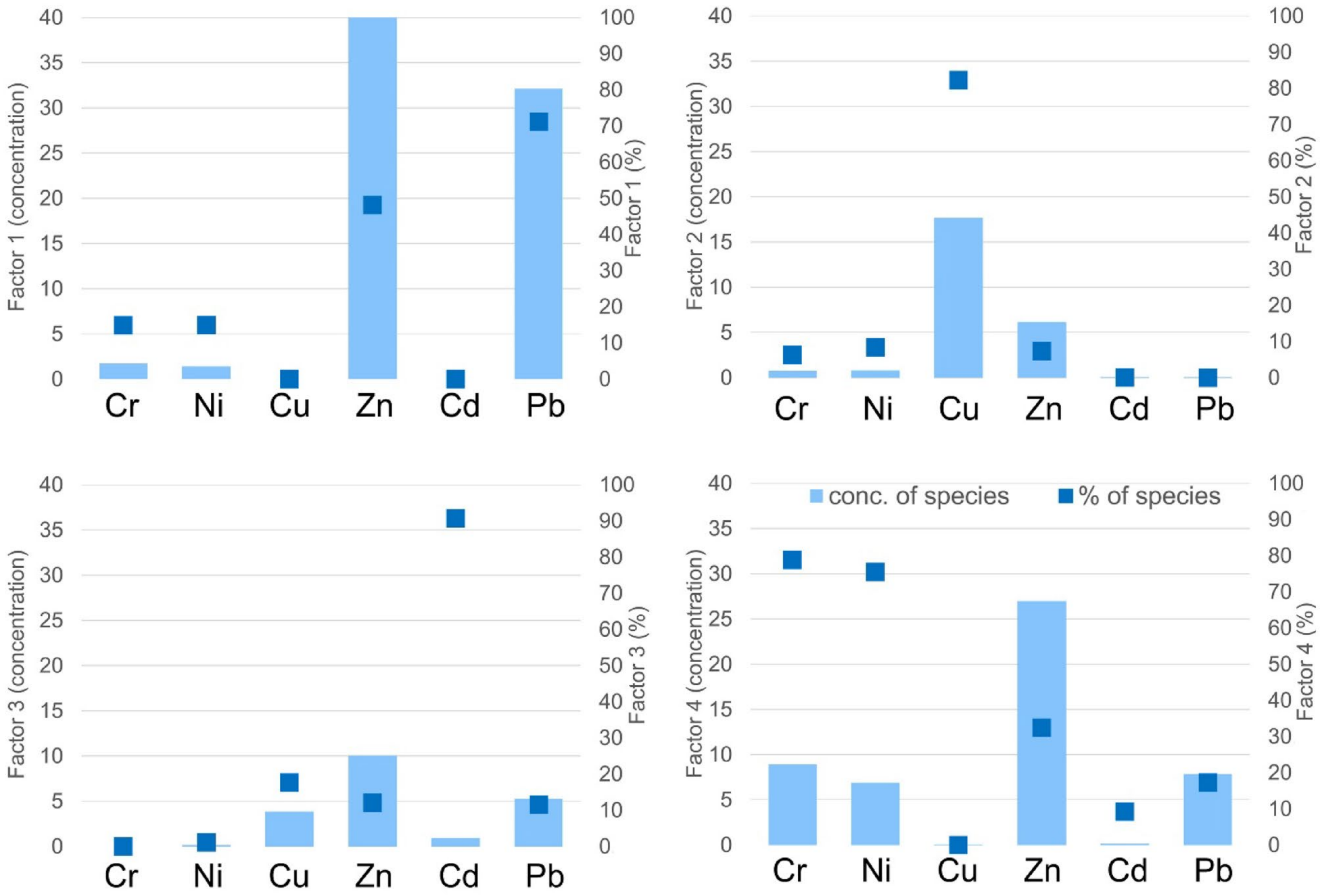
Identification of sources of HMs delivery by the PMF method.

To qualitatively describe the pollution sources contributing to the delivery of HMs to lake surface sediments, a PMF analysis was performed (see Fig. 10 for details). The analysis distinguished four factors, which respectively contribute 24.9%, 17.4%, 22.2%, and 35.6% to the delivery of HMs to lakes. Factor 1 is associated with the delivery of Pb (71.2%) and Zn (48.1%) and about 15% of Cr and Ni. Factor 2 is mainly connected with the delivery of Cu (82.3%). On the other hand, Factor 3 is mainly connected with Cd (90.7%), and to a lesser extent, Cu (17.7%) as well as Zn (12.1%) and Pb (11.6%). Factor 4, on the other hand, is mainly associated with Cr (78.8%) and Ni (75.5%), as well as Zn (32.5%) and Pb (17.3%). Taking into account that the concentrations of HMs were from 49% (for Cd) to 86% (for Pb) higher than the geochemical background values in the surface sediments of the analyzed lakes, it indicates the delivery of these elements from anthropogenic sources^59–61^. It is difficult to attribute the distinguished factors to a specific source of pollution because the influence of many point and non-point sources overlaps. Taking into account the contribution to the delivery of individual HMs by Factor 1 and Factor 2, these factors should be associated with industrial delivery, whereas Factor 3, by considering the contribution of individual HMs, should be associated with urbanized areas. Taking into account the area and contribution of individual pollutants, Factor 4 should be associated with the surface impact from agricultural sources (main supply associated with fertilizer consumption and wastewater management)^62,63^. On the other hand, the exposed contaminant profiles described in the PMF analysis cannot be attributed to a natural origin with a delivery pattern close to the geochemical background.

## Discussion

HMs contamination of water, sediments and plants is a continuous worldwide issue in both developing and developed countries. HMs are resistant and non-degradable elements that are introduced to the surface waters from natural and anthropogenic sources^64,65^. Despite the fact that the analyzed lakes are located in protected areas, which are specifically protected from environmental impacts of anthropogenic sources, sediments are contaminated with HMs. The concentrations of individual HMs were higher than the geochemical background values from 49% (for Cd) to 86% (for Pb). Similar results were obtained by Bella et al.^66^ for Cape Peloro lakes located in a Sicilian protected area or by Slukovskii et al.^67^ for Lake Ukonlampi in the Republic of Karelia. The results showed that levels of Cd and Pb exceeding the maximum residue levels were found in one sample. El-Amier et al.^68^ reported that in Lake Burullus, sediment contaminations were classified in the order Cd < Co < Pb < Cu < Cr < Zn, while the heavy metals in Lake Chaohu were ranked as follows: Cd < Cu < Pb < Ni < Cr < Zn^69^. The results obtained in this study confirm previous research, and median concentration values of HMs showed that they can be arranged in the increasing order: Cd < Ni < Cr < Cu < Pb < Zn. In turn, Li et al.^69^ observed that Cd and Hg were the major pollutants, while the toxicity of Pb was the highest.

It should be noted that the N2 areas were established in Poland in 2004. Thus, the limited human impact in the areas covered by this form of protection has been effective for less than 20 years, and in the context of heavy metal characteristics (persistence, non-biodegradability), the chemical composition of sediments may reflect the state before legal protection of these areas was implemented.

The assessment of HMs pollution of lake surface sediments based on the *I*_*geo*_ showed that the greatest pollution concerns Cu and Pb. In the case of Cu and Pb, *I*_*geo*_ values higher than 2 occurred in 14 and 22 lakes, respectively. The combined assessment of surface sediment contamination based on PLI indicates that the sediments were not polluted with HMs only in 25% of the lakes. Güzel et al.^70^ found that slight and moderate concentration of heavy metals, such as Pb and Zn, were present in most of the sampling points according to PLI and *I*_*geo*_ values. Accordingly, to recognize the amount of anthropogenic input, the enrichment factor (EF) was calculated for the studied heavy metals. In this study, EF indicates that the highest contamination of lake sediments is due to Pb and Cu. In turn, El-Amier^68^ observed that according to the EF value, sediment contamination was in the following order: Cd > Pb > Zn > Co > Cu > Cr. This indicates that Cd is the most enriched and abundant element from anthropogenic activities including chemical fertilizers and untreated wastewater from industrial and agricultural drains^68^.

HMs that have accumulated in sediments can have toxic effects on aquatic biota only in single lakes. The toxic effect in this study is mainly due to concentrations of Cu, Cd, and Pb that are higher than PEC values. Yu et al.^71^ reported that the sediments from Lake Erhai had moderate eco-risk relating to Cu and Pb, and high eco-risk from Cr (73% > PEL), Ni (100% > PEL) and As (93% > PEL). In turn, Güzel et al.^70^ observed that the concentrations of Cr, Cu and especially Ni and Zn pose a significant toxic risk to aquatic organisms in lake sediments of Istanbul in Turkey.

In this study, the identified hot spots for all HMs indicate the dominant influence of point sources (local) of pollution. The analysis showed a different pattern of distribution of Cr (dispersed), Cd and Cu (random), and Ni, Cd, and Pb (clustered) concentrations in lake sediments. Li et al.^26^ analyzed contamination by HMs (Cu, Pb, Zn, Ni, Cr, As, Hg, and Cd) in 190 lakes distributed across five geographic zones in China and observed that industrial sources were the dominant source of heavy metals in lake sediment. In turn, Yu et al.^71^ suggested that narrow spatial variations of anthropogenic Cd, Pb and Zn contaminations show that sediments were influenced by non-point sources. High concentrations of Cd, Pb and Zn were primarily distributed in the southern part of Lake Erhai, which might be due to the input of domestic sewage and industrial wastewater from Dali City and Xiaguan Town. Li et al.^26^ suggested that spatial dispersion of HMs might help to identify pollution sources: Cu and Zn mainly originated from traffic sources, Cd originated from agricultural activities, Pb was related to industrial activities, while Ni and Cr were from mixed sources.

On the basis of the PMF method, it was observed that the contribution to the delivery of individual HMs is affected by four factors. Factors 1 and 2 should be associated with industrial delivery, Factor 3 should be associated with urbanized areas and Factor 4 is associated with the surface impact from agricultural sources (main supply associated with fertilizer consumption and wastewater management). The analysis distinguished four factors, which respectively contribute 24.9%, 17.4%, 22.2%, and 35.6% to the delivery of HMs to lakes. According to Guo et al. (2021), the main pollution of Pb and Zn is from industry-related emission sources. It confirms the results obtained in this study, where pollution sources for Pb and Zn were associated with industrial delivery (Factors 1 and 2)^72^. Factor 4 as a main source of pollution is associated with Cr (78.8%) and Ni (75.5%), as well as Zn (32.5%) and Pb (17.3%). Similar observations were obtained by Guo et al.^72^, where the PMF method showed that the main source of pollution of Cd, Zn, Pb and Cr is agriculture. Cattle slurry, which contains significant amounts of Cd and Zn, is often used as an agricultural fertilizer, while Zn is fed to cattle as a trace metal supplement for growth promotion. Additionally, long-term use of P fertilizer will result in the accumulation of Cd, as well as Pb and Cr, which also occur at high levels in fertilizers^72^.

The present results do not reveal a clear relationship between HMs concentrations in lake sediments and basic land use classes. Lakes are most often supplied by rivers that flow through areas with mixed land cover. Therefore, direct correlation between particular forms of land use/cover properties and the content of HMs is impossible to determine^7^. Further studies related to assessment of the influence of catchment land use and use of land directly adjacent to the lakes should include the analysis of a larger number of land use classes and land use configuration. The first such analyses have already been performed by Mohammadi et al.^73^ and Miranda et al.^74^, who, beside the identification of land use classes themselves, also determined land use configuration. These results obtained for single catchments can provide a starting point for broader analyses. Furthermore, it seems crucial to include additional information on land relief and its configuration in subsequent analyses of land cover and its configuration. Moreover, it should be noted that the study sites are located in the deepest points of the lakes. This location of sampling sites was intended to reduce the impact of point sources of pollution from adjacent areas. In addition, the lakes are located within the range of protected areas, thus the results presented in this paper show more the influence of processes taking place in the entire catchment.

## Conclusions

The results of the multicriteria and multivariate analyses presented in this paper showed that the studied lakes’ surface sediments are contaminated with HMs. The concentrations of individual HMs were higher than the geochemical background values by 49% (for Cd) to 86% (for Pb). Additionally, the values of *I*_*geo*_ and EF indicate that the highest contamination of lake surface sediments is due to Pb and Cu, PLI indicates that only in 25% of the lakes sediments were not polluted. Analysis showed that HMs can have toxic effects on aquatic biota only in single lakes. The toxic effect is mainly due to concentrations of Cu, Cd, and Pb that are higher than PEC values.

Cluster analysis allowed lakes to be divided into four groups. The group of lakes with the highest contamination of all HMs is generally found in central-western Poland. The analysis showed a different pattern of distribution of Cr (dispersed), Cd and Cu (random), and Ni, Cd, and Pb (clustered) concentrations in lake surface sediments, which indicates a different dominant pollution source. The identified hot spots for all HMs indicate the dominant influence of point sources (local) of pollution. The limiting factors for surface sediment pollution of HMs are the presence of wetlands and protected areas, i.e., national parks and nature reserves in the catchment. PMF analysis revealed the presence of diverse and overlapping pollution sources of industrial, urbanized, and agricultural nature.

Analysis of lake and catchment parameters showed that lakes with a larger surface area were less contaminated with HMs. Moreover, there was a decreasing gradient of surface sediment contamination from the west to the east of Poland. The study shows that flow-through lakes with high drainage ratio values had lower Ni and Cr concentrations.

## Supplementary Information

Supplementary Information.

## Data Availability

Data are available upon request from the corresponding author.
