# Supplementary material for: Heavy metals in lake surface sediments in protected areas in Poland: concentration, pollution, ecological risk, sources and spatial distribution
Source: Sci Rep. 2022 Sep 2;12:15006. doi: 10.1038/s41598-022-19298-y (PMC9440085; doi:10.1038/s41598-022-19298-y)
Supplement: Supplementary file 1 — Supplementary Information. [file 41598_2022_19298_MOESM1_ESM.docx]

**Table S1. Catchments characteristics** **investigated in the study**

| **Catchment** | **Id** | **CA**  **(km^2^)** | **ArA (%)** | **AgA (%)** | **FoA (%)** | **WeA (%)** | **WaA**  **(%)** | **DrD**  **(km/km^2^)** | **PrA**  **(%)** |
| --- | --- | --- | --- | --- | --- | --- | --- | --- | --- |
| Babięty Wielkie | 1 | 105.60 | 1.29 | 66.89 | 23.35 | 1.18 | 7.39 | 0.30 | 53.93 |
| Będgoszcz | 2 | 305.01 | 3.86 | 79.41 | 16.67 | 0.00 | 1.82 | 0.42 | 31.86 |
| Białe-Miałkie | 3 | 159.75 | 3.85 | 46.93 | 49.27 | 0.47 | 1.83 | 0.34 | 51.64 |
| Białkowskie | 4 | 29.23 | 0.00 | 52.95 | 47.10 | 0.00 | 5.04 | 0.27 | 88.14 |
| Bierzwnik | 5 | 17.45 | 0.16 | 16.44 | 83.42 | 0.00 | 11.88 | 0.00 | 100.02 |
| Binowskie | 6 | 29.31 | 5.93 | 69.56 | 24.44 | 0.00 | 2.15 | 0.21 | 84.51 |
| Boruja Duża | 7 | 7.71 | 2.97 | 26.53 | 70.62 | 0.00 | 7.66 | 0.00 | 95.35 |
| Budzisławskie | 8 | 28.07 | 1.61 | 80.18 | 18.33 | 0.00 | 4.95 | 0.40 | 54.31 |
| Bukowskie | 9 | 12.88 | 5.48 | 86.11 | 8.42 | 0.00 | 7.74 | 0.19 | 88.34 |
| Bysławskie | 10 | 18.10 | 1.75 | 61.11 | 37.28 | 0.00 | 4.51 | 0.29 | 99.09 |
| Chłopowo | 11 | 65.85 | 1.67 | 43.17 | 55.16 | 0.00 | 2.29 | 0.14 | 94.79 |
| Długie Wigierskie | 12 | 72.75 | 0.50 | 22.52 | 76.95 | 0.36 | 32.84 | 0.10 | 99.96 |
| Dłużec | 13 | 127.15 | 2.85 | 64.25 | 32.83 | 0.00 | 4.90 | 0.25 | 22.33 |
| Dominickie | 14 | 21.28 | 7.68 | 25.59 | 66.79 | 0.00 | 15.52 | 0.00 | 100.05 |
| Drawsko | 15 | 173.98 | 3.26 | 54.02 | 42.77 | 0.20 | 13.73 | 0.17 | 97.06 |
| Dubie Południowe | 16 | 87.43 | 1.90 | 43.48 | 54.65 | 0.00 | 1.66 | 0.29 | 100.02 |
| Dybrzk | 17 | 1747.92 | 2.13 | 31.02 | 66.96 | 0.06 | 4.89 | 0.27 | 52.97 |
| Glinna | 18 | 14.22 | 4.28 | 39.57 | 56.09 | 0.00 | 5.79 | 0.26 | 99.94 |
| Głebokie | 19 | 5.97 | 0.00 | 0.00 | 100.10 | 0.00 | 16.62 | 0.66 | 100.12 |
| Gremzdel | 20 | 15.46 | 0.00 | 87.53 | 12.43 | 0.00 | 6.07 | 0.35 | 12.70 |
| Gwiazda | 21 | 43.11 | 3.18 | 48.33 | 48.62 | 0.00 | 7.26 | 0.37 | 100.13 |
| Kaleńskie | 22 | 15.30 | 0.00 | 29.71 | 70.34 | 0.00 | 18.70 | 0.00 | 98.34 |
| Kaliszańskie | 23 | 17.86 | 0.00 | 79.85 | 20.24 | 0.00 | 20.24 | 0.00 | 22.66 |
| Kamienny Most | 24 | 14.85 | 5.78 | 67.45 | 26.78 | 0.00 | 4.32 | 0.64 | 100.01 |
| Karaś | 25 | 46.27 | 5.84 | 45.09 | 49.21 | 7.60 | 6.46 | 0.00 | 17.85 |
| Kopań | 26 | 37.91 | 3.90 | 65.73 | 30.45 | 3.86 | 19.49 | 0.12 | 27.95 |
| Kosino | 27 | 31.67 | 3.15 | 75.12 | 21.73 | 0.00 | 4.91 | 0.21 | 95.24 |
| Krępsko Długie | 28 | 10.66 | 0.00 | 0.00 | 100.07 | 0.00 | 6.96 | 0.46 | 100.07 |
| Krzemień | 29 | 86.41 | 3.46 | 32.83 | 63.72 | 0.00 | 11.08 | 0.21 | 95.91 |
| Krzywe Dębsko | 30 | 38.77 | 2.43 | 19.78 | 77.82 | 0.00 | 5.66 | 0.18 | 26.83 |
| Kubek | 31 | 8.99 | 0.00 | 0.00 | 100.04 | 0.00 | 7.67 | 0.00 | 100.04 |
| Liptowskie | 32 | 71.21 | 0.73 | 29.84 | 69.49 | 0.00 | 3.46 | 0.00 | 79.78 |
| Lubosz Wielki | 33 | 12.56 | 2.10 | 67.47 | 30.49 | 0.00 | 11.80 | 0.00 | 28.29 |
| Marta | 34 | 18.03 | 0.00 | 13.84 | 86.22 | 0.00 | 4.26 | 0.00 | 100.05 |
| Mokre | 35 | 597.06 | 1.43 | 40.32 | 58.34 | 0.90 | 8.23 | 0.31 | 75.05 |
| Morzycko | 36 | 62.55 | 3.49 | 58.47 | 37.95 | 0.00 | 7.48 | 0.20 | 99.91 |
| Niedzięgiel | 37 | 42.21 | 1.11 | 46.71 | 52.31 | 2.22 | 13.05 | 0.00 | 92.55 |
| Nowa Korytnica | 38 | 181.68 | 4.15 | 17.35 | 78.54 | 0.00 | 1.68 | 0.29 | 20.82 |
| Obłęże | 39 | 28.58 | 0.00 | 23.49 | 76.60 | 0.00 | 2.08 | 0.13 | 18.33 |
| Ocypel Wielki | 40 | 26.84 | 1.68 | 7.77 | 90.69 | 0.00 | 4.27 | 0.12 | 100.13 |
| Piaseczno | 41 | 9.24 | 2.34 | 46.37 | 51.24 | 0.00 | 6.27 | 0.00 | 48.00 |
| Piaseczno Duże | 42 | 11.11 | 0.00 | 15.40 | 84.64 | 0.00 | 11.05 | 0.19 | 100.04 |
| Płaskie | 43 | 5.60 | 0.00 | 0.00 | 99.93 | 0.00 | 10.17 | 0.00 | 99.93 |
| Pławno | 44 | 9.02 | 1.67 | 66.30 | 32.09 | 0.00 | 6.57 | 0.00 | 80.74 |
| Płociczno | 45 | 286.60 | 1.12 | 39.18 | 59.75 | 0.12 | 2.71 | 0.17 | 75.90 |
| Postne | 46 | 15.23 | 0.00 | 39.15 | 60.80 | 0.00 | 2.99 | 0.00 | 92.10 |
| Przemęckie Zachodnie | 47 | 183.07 | 5.12 | 47.24 | 47.69 | 0.41 | 3.78 | 0.32 | 57.50 |
| Przytoczno | 48 | 82.41 | 1.13 | 21.52 | 77.38 | 0.00 | 4.90 | 0.30 | 100.02 |
| Przytoń | 49 | 17.84 | 1.41 | 74.24 | 24.38 | 0.00 | 6.04 | 0.06 | 95.71 |
| Resko | 50 | 2.00 | 0.00 | 52.59 | 47.48 | 0.00 | 26.77 | 0.00 | 100.02 |
| Rudno | 51 | 1265.30 | 2.85 | 35.84 | 61.35 | 0.20 | 5.81 | 0.28 | 71.64 |
| Sarbsko | 52 | 187.59 | 1.98 | 45.80 | 52.35 | 0.34 | 3.26 | 0.39 | 34.72 |
| Serwy | 53 | 58.64 | 1.71 | 15.45 | 82.80 | 0.00 | 8.06 | 0.06 | 99.96 |
| Sianowskie | 54 | 67.35 | 2.58 | 65.95 | 31.60 | 0.00 | 6.56 | 0.33 | 100.03 |
| Sierakowo | 55 | 15.18 | 2.58 | 47.91 | 49.51 | 0.00 | 6.11 | 0.44 | 91.06 |
| Sitno | 56 | 233.34 | 1.37 | 45.74 | 52.94 | 0.15 | 2.23 | 0.16 | 70.39 |
| Skąpe | 57 | 31.49 | 2.63 | 32.70 | 64.81 | 0.00 | 10.03 | 0.27 | 100.13 |
| Skotawsko Wielkie | 58 | 8.09 | 0.00 | 8.78 | 91.35 | 0.00 | 8.70 | 0.84 | 100.11 |
| Skulska Wieś | 59 | 59.77 | 2.88 | 81.98 | 15.28 | 0.94 | 2.01 | 0.26 | 27.96 |
| Strzeszyno | 60 | 81.12 | 1.68 | 22.97 | 75.41 | 0.71 | 9.24 | 0.07 | 95.81 |
| Szerokie | 61 | 8.79 | 0.00 | 0.00 | 100.04 | 0.00 | 11.80 | 0.28 | 65.21 |
| Śremskie | 62 | 17.30 | 0.98 | 70.12 | 28.94 | 0.00 | 6.34 | 0.64 | 85.72 |
| Świętajno | 63 | 17.80 | 2.22 | 8.15 | 89.71 | 0.66 | 9.80 | 0.40 | 100.09 |
| Tarczyńskie | 64 | 315.05 | 2.19 | 66.12 | 31.81 | 0.38 | 5.16 | 0.32 | 47.79 |
| Trzebuń | 65 | 78.19 | 0.68 | 14.41 | 84.93 | 2.11 | 5.69 | 0.31 | 33.42 |
| Tuczno | 66 | 127.51 | 1.53 | 42.76 | 55.76 | 0.28 | 2.95 | 0.10 | 71.08 |
| Tuczno | 67 | 49.13 | 3.02 | 46.80 | 50.21 | 0.69 | 4.93 | 0.33 | 14.08 |
| Ustrych | 68 | 442.93 | 2.42 | 34.27 | 63.43 | 0.72 | 8.15 | 0.29 | 52.55 |
| Wełtyńskie | 69 | 15.55 | 1.59 | 40.49 | 57.84 | 2.37 | 17.80 | 0.00 | 70.12 |
| Wielkie | 70 | 48.57 | 1.62 | 67.99 | 30.45 | 0.00 | 7.01 | 0.32 | 42.44 |
| Wielkie Dąbie | 70 | 850.45 | 3.67 | 43.17 | 53.20 | 0.04 | 7.48 | 0.26 | 86.42 |
| Wierzbiczańskie | 72 | 38.98 | 3.74 | 68.69 | 27.69 | 0.00 | 3.90 | 0.26 | 12.08 |
| Wilczyńskie | 73 | 32.57 | 4.90 | 80.01 | 15.22 | 0.00 | 5.05 | 0.38 | 65.37 |
| Wukśniki | 74 | 4.75 | 0.00 | 60.83 | 39.31 | 0.00 | 25.91 | 0.00 | 58.26 |
| Załom Wielki | 75 | 105.19 | 1.75 | 26.17 | 72.14 | 0.00 | 2.05 | 0.25 | 91.70 |
| Zarybinek | 76 | 275.64 | 2.41 | 68.47 | 29.25 | 0.43 | 5.11 | 0.33 | 41.19 |
| Żerdno | 77 | 69.53 | 1.12 | 50.50 | 48.44 | 0.51 | 3.82 | 0.27 | 100.06 |

**Table S2. Lake characteristics investigated in the study**

| **Lake** | **Id** | **Lon** | **Lat** | **LA**  **[ha]** | **LV**  **[mln m^3^]** | **AvD**  **[m]** | **MaD**  **[m]** | **WsE**  **[m asl]** | **Hydrological type** | **SDI** | **LDR** |
| --- | --- | --- | --- | --- | --- | --- | --- | --- | --- | --- | --- |
| Babięty Wielkie | 1 | 21.12227 | 53.71479 | 250.40 | 59.90 | 23.90 | 652.00 | 140.00 | FT | 2.30 | 42.17 |
| Będgoszcz | 2 | 14.81482 | 53.23479 | 264.00 | 15.80 | 5.50 | 13.00 | 14.00 | FT | 3.25 | 115.53 |
| Białe-Miałkie | 3 | 16.22528 | 51.93476 | 104.00 | 2.00 | 1.90 | 10.20 | 61.00 | FT | 1.48 | 153.61 |
| Białkowskie | 4 | 16.23153 | 52.56977 | 145.90 | 14.00 | 9.60 | 31.40 | 83.00 | FT | 2.57 | 20.03 |
| Bierzwnik | 5 | 15.62163 | 53.02573 | 205.20 | 11.20 | 5.40 | 12.40 | 69.00 | Ex | 2.84 | 8.50 |
| Binowskie | 6 | 14.63599 | 53.31015 | 52.40 | 2.90 | 5.50 | 9.40 | 40.00 | En | 1.63 | 55.94 |
| Boruja Duża | 7 | 17.43995 | 54.09303 | 65.10 | 3.30 | 5.10 | 10.30 | 65.00 | Ex | 2.21 | 11.85 |
| Budzisławskie | 8 | 18.05309 | 52.46132 | 155.90 | 17.20 | 11.10 | 36.70 | 99.00 | FT | 2.23 | 18.01 |
| Bukowskie | 9 | 15.55582 | 53.08702 | 60.60 | 5.30 | 8.80 | 34.40 | 73.00 | FT | 2.46 | 21.25 |
| Bysławskie | 10 | 17.99212 | 53.51399 | 69.00 | 7.00 | 10.20 | 28.50 | 98.00 | FT | 2.31 | 26.24 |
| Chłopowo | 11 | 15.53345 | 53.04817 | 72.50 | 7.90 | 10.90 | 27.90 | 76.00 | En | 1.48 | 90.82 |
| Długie Wigierskie | 12 | 23.02757 | 54.0241 | 80.00 | 5.20 | 6.40 | 14.80 | 131.00 | En | 2.43 | 90.94 |
| Dłużec | 13 | 14.6597 | 53.09021 | 85.20 | 5.20 | 6.10 | 10.40 | 46.00 | FT | 1.77 | 149.23 |
| Dominickie | 14 | 16.31 | 51.95066 | 343.90 | 22.20 | 6.50 | 17.10 | 61.00 | Ex | 1.63 | 6.19 |
| Drawsko | 15 | 16.17781 | 53.59364 | 1871.50 | 331.40 | 17.70 | 82.20 | 128.00 | FT | 4.84 | 9.30 |
| Dubie Południowe | 16 | 15.74433 | 53.20915 | 112.00 | 8.10 | 7.30 | 34.40 | 77.00 | FT | 1.83 | 78.06 |
| Dybrzk | 17 | 17.60694 | 53.84541 | 216.50 | 18.90 | 8.80 | 19.00 | 119.00 | FT | 1.97 | 807.35 |
| Glinna | 18 | 14.68556 | 53.29052 | 75.60 | 6.20 | 8.30 | 16.40 | 25.00 | FT | 2.38 | 18.81 |
| Głebokie | 19 | 17.38914 | 54.27578 | 107.50 | 12.30 | 11.50 | 31.20 | 91.00 | FT | 2.25 | 5.55 |
| Gremzdel | 20 | 23.16715 | 54.14375 | 59.30 | 1.90 | 3.20 | 10.00 | 142.00 | FT | 2.21 | 26.07 |
| Gwiazda | 21 | 17.96436 | 53.53103 | 53.00 | 3.80 | 7.20 | 20.20 | 98.00 | FT | 2.48 | 81.34 |
| Kaleńskie | 22 | 16.17777 | 53.52973 | 106.20 | 12.20 | 11.50 | 33.70 | 140.00 | En | 2.74 | 14.41 |
| Kaliszańskie | 23 | 17.12966 | 52.88662 | 297.20 | 26.00 | 8.80 | 26.90 | 64.00 | Ex | 1.68 | 6.01 |
| Kamienny Most | 24 | 15.39026 | 53.46737 | 58.10 | 0.50 | 1.00 | 2.00 | 76.00 | FT | 2.20 | 25.55 |
| Karaś | 25 | 19.48213 | 53.55459 | 423.30 | 2.60 | 0.60 | 4.50 | 98.00 | Ex | 2.00 | 10.93 |
| Kopań | 26 | 16.45016 | 54.48197 | 789.70 | 14.70 | 1.90 | 3.90 | 0.10 | Ex | 1.50 | 4.80 |
| Kosino | 27 | 15.59479 | 53.06692 | 50.00 | no data | no data | no data | 66.00 | FT | 1.53 | 63.33 |
| Krępsko Długie | 28 | 16.60361 | 53.37249 | 73.90 | 5.60 | 7.60 | 15.10 | 84.00 | FT | 1.88 | 14.42 |
| Krzemień | 29 | 15.53888 | 53.37593 | 229.10 | 21.90 | 9.60 | 29.20 | 91.00 | FT | 1.62 | 37.72 |
| Krzywe Dębsko | 30 | 15.88084 | 53.23178 | 121.60 | 7.10 | 5.90 | 1.10 | 79.00 | FT | 2.35 | 31.88 |
| Kubek | 31 | 16.07648 | 52.69392 | 69.00 | 1.30 | 2.00 | 3.50 | 42.00 | En | 2.36 | 13.02 |
| Liptowskie | 32 | 16.18439 | 53.18506 | 134.90 | 12.40 | 9.20 | 29.00 | 82.00 | Ex | 1.75 | 52.78 |
| Lubosz Wielki | 33 | 16.1907 | 52.51223 | 94.00 | 8.70 | 9.40 | 29.20 | 94.00 | Ex | 1.55 | 13.37 |
| Marta | 34 | 16.05991 | 53.17593 | 661.00 | 5.10 | 7.70 | 25.00 | 72.00 | En | 0.75 | 2.73 |
| Mokre | 35 | 21.38783 | 53.68245 | 841.00 | 107.30 | 12.70 | 51.00 | 124.00 | FT | 2.48 | 70.99 |
| Morzycko | 36 | 14.40657 | 52.86325 | 342.00 | 49.80 | 14.50 | 60.00 | 51.00 | FT | 1.72 | 18.29 |
| Niedzięgiel | 37 | 17.88875 | 52.44741 | 637.70 | 35.10 | 5.50 | 21.60 | 104.00 | Ex | 2.65 | 6.62 |
| Nowa Korytnica | 38 | 15.98862 | 53.21799 | 111.30 | 2.60 | 2.40 | 4..7 | 77.00 | FT | 1.97 | 163.24 |
| Obłęże | 39 | 16.91723 | 54.24302 | 62.40 | 3.70 | 6.00 | 8.90 | 34.00 | FT | 1.37 | 45.80 |
| Ocypel Wielki | 40 | 18.30472 | 53.80016 | 114.00 | 8.10 | 6.70 | 40.00 |  | FT | 1.82 | 23.54 |
| Piaseczno | 41 | 14.74551 | 53.07573 | 52.00 | no data | no data | no data | 75.00 | Ex | 1.71 | 17.77 |
| Piaseczno Duże | 42 | 16.00821 | 53.12832 | 58.70 | 4.50 | 7.60 | 25.90 | 62.00 | En | 1.43 | 18.93 |
| Płaskie | 43 | 23.41465 | 53.91312 | 56.80 | 2.80 | 5.00 | 12.00 | 113.00 | En | 1.03 | 9.87 |
| Pławno | 44 | 16.21856 | 53.53867 | 52.90 | 2.00 | 3.80 | 7.40 | 140.00 | En | 2.22 | 17.05 |
| Płociczno | 45 | 15.99945 | 53.1347 | 56.10 | 1.50 | 2.70 | 5.20 | 59.00 | FT | 1.61 | 510.88 |
| Postne | 46 | 14.84366 | 52.78739 | 51.40 | 0.90 | 1.80 | 3.30 | 46.00 | Ex | 1.67 | 29.63 |
| Przemęckie Zachodnie | 47 | 16.19575 | 51.94508 | 220.00 | 7.00 | 3.20 | 5.60 | 60.00 | FT | 1.41 | 83.21 |
| Przytoczno | 48 | 15.75358 | 53.07493 | 227.60 | 10.30 | 4.50 | 12.50 | 71.00 | FT | 2.74 | 36.21 |
| Przytoń | 49 | 15.86347 | 53.63748 | 109.70 | 8.40 | 7.70 | 20.30 | 121.00 | En | 1.76 | 16.26 |
| Resko | 50 | 15.96558 | 53.67741 | 50.70 | 1.30 | 2.70 | 5.00 | 145.00 | En | 2.47 | 3.94 |
| Rudno | 51 | 15.76555 | 53.22315 | 64.50 | no data | no data | no data |  | FT | 1.98 | 1961.71 |
| Sarbsko | 52 | 17.62729 | 54.76463 | 651.00 | 8.00 | 1.20 | 3.20 | 0.50 | FT | 1.72 | 28.82 |
| Serwy | 53 | 23.20902 | 53.91471 | 460.30 | 67.10 | 14.10 | 41.50 | 126.00 | FT | 2.64 | 12.74 |
| Sianowskie | 54 | 18.08717 | 54.37894 | 71.00 | no data | no data | no data | 140.50 | FT | 1.42 | 94.86 |
| Sierakowo | 55 | 15.46694 | 53.31087 | 64.80 | 3.50 | 5.50 | 11.70 | 70.00 | FT | 1.27 | 23.42 |
| Sitno | 56 | 16.02787 | 53.18563 | 67.20 | 2.60 | 4.00 | 7.00 | 69.00 | FT | 1.41 | 347.24 |
| Skąpe | 57 | 17.81297 | 53.90425 | 132.20 | 9.40 | 7.10 | 20.10 | 139.00 | FT | 3.00 | 23.82 |
| Skotawsko Wielkie | 58 | 17.53398 | 54.28053 | 80.00 | 3.20 | 4.10 | 8.70 | 108.00 | FT | 2.44 | 10.11 |
| Skulska Wieś | 59 | 18.31783 | 52.49194 | 120.10 | 8.00 | 6.70 | 17.60 | 86.00 | FT | 1.92 | 49.77 |
| Strzeszyno | 60 | 16.42972 | 53.61873 | 63.10 | 2.70 | 4.30 | 12.40 | 130.00 | FT | 1.74 | 128.56 |
| Szerokie | 61 | 15.91425 | 53.2196 | 76.30 | 4.60 | 6.10 | 15.80 | 80.00 | FT | 1.73 | 11.52 |
| Śremskie | 62 | 16.049 | 52.60896 | 117.60 | 23.70 | 20.20 | 45.00 | 39.00 | FT | 1.18 | 14.71 |
| Świętajno | 63 | 21.24243 | 53.6037 | 213.00 | 25.40 | 11.90 | 29.50 | 132.00 | FT | 2.26 | 8.36 |
| Tarczyńskie | 64 | 19.88686 | 53.34595 | 163.80 | 6.10 | 3.80 | 9.20 | 143.00 | FT | 1.79 | 192.34 |
| Trzebuń | 65 | 15.72524 | 53.30695 | 136.00 | 12.50 | 9.20 | 20.00 | 80.00 | FT | 2.24 | 57.50 |
| Tuczno | 66 | 16.12516 | 53.19339 | 128.90 | 11.60 | 9.10 | 20.20 | 68.00 | FT | 1.50 | 98.92 |
| Tuczno | 67 | 15.85183 | 52.58872 | 51.90 | 8.10 | 15.70 | 37.80 | 43.00 | FT | 1.49 | 94.67 |
| Ustrych | 68 | 20.49732 | 53.636 | 93.10 | 5.10 | 5.50 | 11.60 | 93.00 | FT | 1.91 | 475.75 |
| Wełtyńskie | 69 | 14.58004 | 53.23872 | 310.10 | 13.90 | 4.10 | 11.60 | 26.00 | Ex | 2.62 | 5.01 |
| Wielkie | 70 | 16.25997 | 52.64419 | 260.80 | 25.00 | 8.50 | 30.10 | 46.00 | FT | 1.86 | 18.62 |
| Wielkie Dąbie | 71 | 15.86413 | 53.39969 | 91.40 | 3.80 | 4.20 | 8.10 | 89.00 | FT | 1.80 | 930.47 |
| Wierzbiczańskie | 72 | 17.73112 | 52.52856 | 189.30 | 12.70 | 6.70 | 21.60 | 97.00 | FT | 2.27 | 20.59 |
| Wilczyńskie | 73 | 18.11118 | 52.48029 | 189.50 | 14.70 | 7.80 | 24.90 | 99.00 | FT | 2.98 | 17.19 |
| Wukśniki | 74 | 20.09751 | 53.97004 | 117.10 | 27.30 | 23.40 | 67.30 | 113.00 | En | 1.51 | 4.05 |
| Załom Wielki | 75 | 16.06212 | 53.08503 | 104.70 | 5.60 | 5.40 | 21.50 | 60.00 | FT | 2.29 | 100.46 |
| Zarybinek | 76 | 19.94829 | 53.37435 | 73.80 | 1.70 | 2.40 | 7.00 | 73.00 | FT | 1.23 | 373.49 |
| Żerdno | 77 | 16.21578 | 53.60598 | 205.00 | 31.20 | 15.20 | 36.00 | 128.00 | FT | 1.46 | 33.92 |

**Table S3. List of geochemical indices used in this study**

| **Index** |  | **Formula** | **Classification** | **Source** |
| --- | --- | --- | --- | --- |
| Geoaccumulation Index | *I_geo_* | *I_geo_* = log_2_[Ci/1.5Bi]  where:  Ci - the concentration of HM in the sediments (mg·kg^-1^),  Bi - the reference geochemical background value of each HM | Class 0:  *I_geo_* ≤0 no enrichment,  Class 1: 0< *I_geo_* ≤1 minor enrichment,  Class 2: 1<  *I_geo_* ≤2 moderate enrichment,  Class 3: 2<  *I_geo_* ≤3 moderately severe enrichment,  Class 4: 3<  *I_geo_* ≤4 severe enrichment,  Class 5: 4<  *I_geo_* ≤5 very severe enrichment,  Class 6:  *I_geo_* >5 extremely severe enrichment | Muller (1969)  Muller (1981) |
| Enrichment Factor | EF | EFi = (Ci/CFe) / (Bi/BFe)  where:  Ci - the concentration of HM in the sediments (mg·kg^-1^),  CFe - the concentration of iron (Fe)  Bi - the reference geochemical background value of each HM  BFe - the reference geochemical background value of iron (Fe) | Class 0: EF≤1 no enrichment,  Class 1: 1<EF≤3 minor enrichment,  Class 2: 3<EF≤5 moderate enrichment,  Class 3: 5<EF≤10 moderately severe enrichment,  Class 4: 10<EF≤25 severe enrichment,  Class 5: 25<EF≤50 very severe enrichment,  Class 6: EF>50 extremely severe enrichment | Ergin et al. (1991) |
| Contamination Factor | CF | CF_i_ = C_i_/B_i_  where:  Ci - the concentration of HM in the sediments (mg·kg^-1^),  Bi - the reference geochemical background value of each HM | CF < 1 low,  1≤CF < 3 moderate,  3≤CF < 6 considerable,  CF≥6 very high | Martin and Meybeck (1979) |
| Pollution Load Index | PLI | PLI = (CF_i1_× CF_i2_ × … × CF_in_)^1/n^  where:  n - the number of HMs,  CF - contamination factor defined for each studied HM | PLI < 1 no pollution,  PLI ≥ 1 pollution | Tommilson et al. (1980)  Harikumar et al. (2009) |
| Metal Pollution Index | MPI | MPI = (C_i1_× C_i2_ × … × C_in_)^1/n^  where:  C_i_ - the concentration of HM in the sediments (mg·kg^-1^),  n - the number of considered HMs | MPI < 1 no pollution,  MPI ≥ 1 pollution | Usero et al. (1997) |
| Toxic Risk Index | TRI | TRI = ∑*n_i_*_=1_TRI_i_ = {[(C_i_/TEC_i_)^2^+(C_i_/PEC_i_)^2^]/2}^1/2^  where:  n - the number of HMs,  C_i_ - the concentration of HM in the sediments (mg·kg^-1^),  TEC_i_ - the threshold effect concentration of each HM,  PEC_i_ - the probable effect concentration of each HM,  TRI_i_ - the toxic risk index of each HM | TRI ≤ 5 no toxic risk,  5 < TRI ≤ 10 low,  10 < TRI ≤ 15 moderate,  15 < TRI ≤ 20 considerable,  20 < TRI very high | Zhang et al. (2016)  Bing et al. 2019 |
